# Discovery of time-delayed gene regulatory networks based on temporal gene expression profiling

**DOI:** 10.1186/1471-2105-7-26

**Published:** 2006-01-18

**Authors:** Xia Li, Shaoqi Rao, Wei Jiang, Chuanxing Li, Yun Xiao, Zheng Guo, Qingpu Zhang, Lihong Wang, Lei Du, Jing Li, Li Li, Tianwen Zhang, Qing K Wang

**Affiliations:** 1 Department of Bioinformatics, Harbin Medical University, Harbin 150086, PR China; 2 Departments of Cardiovascular Medicine and Molecular Cardiology, The Cleveland Clinic Foundation, 9500 Euclid Avenue, Cleveland, Ohio 44195, USA; 3 Department of Computer Science, Harbin Institute of Technology, Harbin 150080, PR China; 4 Biomedical Engineering Institute, Capital University of Medical Sciences, Beijing 100054, PR China; 5 Department of Molecular Medicine, Cleveland Clinic Lerner College of Medicine, Case Western Reserve University, 9500 Euclid Avenue, Cleveland, Ohio 44195, USA

## Abstract

**Background:**

It is one of the ultimate goals for modern biological research to fully elucidate the intricate interplays and the regulations of the molecular determinants that propel and characterize the progression of versatile life phenomena, to name a few, cell cycling, developmental biology, aging, and the progressive and recurrent pathogenesis of complex diseases. The vast amount of large-scale and genome-wide time-resolved data is becoming increasing available, which provides the golden opportunity to unravel the challenging reverse-engineering problem of time-delayed gene regulatory networks.

**Results:**

In particular, this methodological paper aims to reconstruct regulatory networks from temporal gene expression data by using delayed correlations between genes, i.e., pairwise overlaps of expression levels shifted in time relative each other. We have thus developed a novel model-free computational toolbox termed TdGRN (Time-delayed Gene Regulatory Network) to address the underlying regulations of genes that can span any unit(s) of time intervals. This bioinformatics toolbox has provided a unified approach to uncovering time trends of gene regulations through decision analysis of the newly designed time-delayed gene expression matrix. We have applied the proposed method to yeast cell cycling and human HeLa cell cycling and have discovered most of the underlying time-delayed regulations that are supported by multiple lines of experimental evidence and that are remarkably consistent with the current knowledge on phase characteristics for the cell cyclings.

**Conclusion:**

We established a usable and powerful model-free approach to dissecting high-order dynamic trends of gene-gene interactions. We have carefully validated the proposed algorithm by applying it to two publicly available cell cycling datasets. In addition to uncovering the time trends of gene regulations for cell cycling, this unified approach can also be used to study the complex gene regulations related to the development, aging and progressive pathogenesis of a complex disease where potential dependences between different experiment units might occurs.

## Background

With the completion of sequencing entire human genomes, the focus of modern biology has gradually shifted to functional genomics [1]. Many important biological processes (e.g., cellular differentiation during development, aging, disease aetiology etc.) are very unlikely controlled by a single gene instead by the underlying complex regulatory interactions between thousands of genes within a four-dimension space [2]. With advance of molecular biology techniques, it has now become possible to measure the gene expression levels (mRNA levels) of most, if not all, of the genes of an organism simultaneously. The goal of this study was to reversely engineer the underlying gene regulation networks of arbitrary time frames from the temporal gene expression profiling, which would greatly expand our knowledge for complex biological process like disease pathogenesis and eventually provide a clear picture of gene life to locate effective drugs or unknown molecular targets [3,4].

The time-delayed gene regulation pattern in organisms is a common phenomenon [2,5] so that it can be conceived that multiple-time delayed gene regulations are the norm and the single-time delayed ones are the exception. For example, there is a gene (say *g*_1_) whose inhibitory effect (say on gene *g*_2_) depends on an inducer (say *g*_3_) that has to be bound first in order to be able to bind to the inhibition site on *g*_2_. Therefore, there can be a significant delay between the expression of the inhibitor gene *g*_1 _and its observed effect, i.e., the inhibition of gene *g*_2_. In addition, in the reconstruction of gene regulatory networks based on the gene expression profiles, there is also a time-delayed phenomenon in gene regulations because not all the genes that influence the expression level of a gene are necessarily observable in one microarray experiment. For instance, assume that genes *g*_1_, *g*_2_, …, *g*_*n *_are the genes under study. Suppose that gene *g*_1 _regulates gene *g*_*u*_. It is quite possible that *g*_*u *_is not among the genes that are being monitored in the experiment, or its function is currently unknown. Suppose that gene *g*_*u *_in turn controls gene *g*_2_. Since the expression of *g*_*u *_is not observed in the expression profiles, there can also be a significant delay between the expression of *g*_1 _and *g*_2_. Even if all the genes are monitored in an experiment, the unknown factor denoted by *g*_*u *_may stand for a non-genetic environmental factor that leads to a delayed gene regulation based on the expression profiles [2]. Furthermore, self-degradation of mRNA or gene product can be modelled as a time-delayed interaction [6] and such a regulatory rule is not identifiable using conventional approaches if priori knowledge of kinetic parameters, particularly the degradation rate constant, is lacking [7].

At present, there are different approaches to gene networking, for example, Boolean models [8-10], Best-fit models [4,11], Bayesian networks [12-15], Genetic algorithm [16,17], Support vector machines [18], Association rules [19], Neural networks [17,20], Tree models [21] and dynamic models [6,22,23]. Generally, most methods only consider the static (at the same time point) gene expression profiles, so they cannot be used to interpret the time-delayed phenomena of gene regulations. Although several authors [21,24] have well recognized the issue for the time-delayed gene regulations, they merely dealt with the gene networks delayed one unit of time (the interval of two time points in one experiment). Using a Boolean model, Silvescu et al. [2] considered the regulation delayed multiple units (*T*) of time apart, but the Boolean model was restricted to logical (Boolean) relationships between variables and relies on the assumption that the expression of a gene is likely to be controlled by a relatively small number (say *k*) of genes [8,9,25]. Nevertheless, such a biologically meaningful value of *k *is often unknown prior to a detailed investigation [2]. Recently, several investigators have addressed genome duplication as the evolution force for creating new genes in genomes and for gene regulatory network growth [26-29].

The exponential increases of the amount of massive time-series data have provided good opportunities to uncover causal gene-gene or gene-phenotype relationships and to characterize the dynamic properties of the underlying molecular networks for various biological processes. Time series data structure offers a necessary (although not sufficient) condition – time lag to infer a cause-effect relationship [30]. However, in order to fully exploit the power and value of computational networking approaches to systematically dissecting the dynamic mechanisms of the intricate molecular interplays, solutions to several significant challenges remain to be solved. Nowadays, several delicately developed dynamic models (for example, Probabilistic Boolean Networks (PBN) [31]; Dynamic Bayesian Networks (DBN) [30]; Hidden Markov Model (HMM) [32] and Kalman filters [33]) for reconstructing longitudinal regulatory networks are model-based in the sense that an explicit mathematical model is required. When the model of the system is unknown, the networking problem becomes intractable. It is perceived that model-based approaches are more powerful to distil the studied biological process and behaviours into a set of compact mathematical equations given that the correct models (e.g. the error model and the network architecture) for the studied complexities can be precisely specified. However, it has been suggested that the results of model-based methods can be compromised if the underlying model is wrongly assumed. An recent study for modelling CDC2/APC network in cell cycling [34] explored the potential of using a cell-cycle oscillator differential-equation model to uncover the role of positive- and negative-feedback loops in the CDC2/APC system. The authors found that there are significant discrepancies between the model-based learnt results and the true networking architectures. The authors thus believe that the cell-cycle periodical oscillator mechanisms are resulted from the synergic actions of both positive and negative feedbacks, which are however hard to be depicted precisely by any mathematical model.

The scientific values of robust model-free approaches (e.g. many data mining algorithms) for probing the unknown space and complexities of dynamic molecular networks have been increasingly recognized. Because most of gene networks are hard to be mapped precisely by any parsimonious mathematical model, data mining approaches, a way to compute control actions directly from the input/output data without first creating any model at all, have received increasing attentions. Remondini et al. [35] studied the dynamics of a gene expression time series network (specifically, the regulatory network for c-Myc-activated genes) based on the correlations of gene expressions. Compared with a linear Markov model, the network built by the model-free method demonstrates global dynamic properties that emerge after cell state perturbation. In this paper, we described a novel model-free approach for reconstructing time-delayed gene regulatory networks. We focused on the dependencies between the activities of genes that span more than one unit of time. The method, called TdGRN (Time-delayed Gene Regulatory Network), allows the expression of a target gene at time *t *+ 1 to be interacted with other genes at time frames {*t*, *t *- 1, …, *t *- (*T *- 1)}. For each target gene, we constructed its time-delayed gene expression profiles. Then, we used a decision tree to discover the time-delayed regulations that modulate the activities of the target gene. In our method, we neither assumed any arbitrary threshold for discretisation nor the definition of the number of regulating genes, *k*, nor the network structure. We uncovered the regulations between genes empirically from the decision tree analysis of temporal gene expression profiles. In order to avoid false positives, we used a conservative method to filter the constructed decision trees. Only trees that had adequate classification accuracy (called confident tree and putative tree; see Methods for definitions) were kept for the following gene networking. We have explored the behaviours and properties of the novel method by analyzing two publicly available datasets for *Saccharomyces cerevisiae *and human HeLa cell cycles, respectively.

## Results

### Description of the two datasets

**Figure 1:**
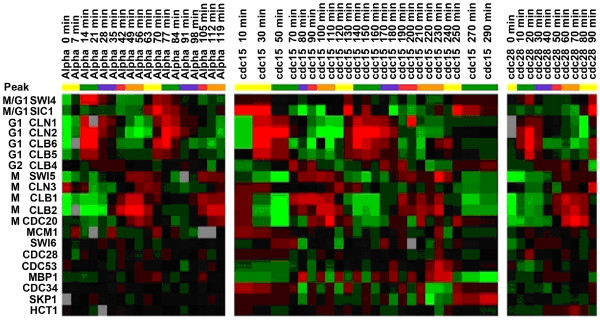
**Gene expression during the yeast cell cycle**. In this figure, each gene corresponds to a row, and the time point for each column is denoted. The magnitude of mRNA expression ratio for each gene is represented by different colours. Red indicates mRNA abundance; green indicates a dearth; and gray indicates absence of the data. The colours at the top bar distinguish the cell cycle phases (M/G1, yellow; G1, green; S, purple; G2, red; M, orange).

We report the results from analysis of two well-known datasets for yeast cells and human HeLa cells, respectively. The yeast cell cycling dataset is from the microarray data analyzed initially by Spellman et al. [36] and Cho et al. [37], who obtained the genome-wide transcriptions from the *Saccharomyces cerevisiae *cell cultures that were synchronized by three different methods: *cdc15*, *cdc28 *and *alpha-factor*. We chose the *cdc15 *synchronized dataset for training and used the remaining datasets (*cdc28 *and *alpha-factor*) for testing. To facilitate the interpretation of underlying time-delayed regulations, we only used adjacent equidistant measurements at the equal units of time (i.e. several time points for the data sets namely *cdc15 *were truncated). Thus, there were 19, 17 and 18 available time points for the synchronizations (*cdc15*, *cdc28 *and *alpha-factor*), respectively (figure 1). All major transitions in the budding yeast cell cycle are regulated by cyclins via the associated cyclin-dependent kinase (CDK) activity [21]. For the purpose of a numeric demonstration, we chose 20 yeast cyclin genes: CLN1, CLN2, CLN3, CLB1, CLB2, CLB4, CLB5, CLB6, CDC28, MBP1, CDC53, CDC34, SKP1, SWI4, SWI5, SWI6, HCT1, CDC20, SIC1 and MCM1. These genes have been known to be involved in the cell-cycle regulations and their temporal activities are exhibited on Figure 1, based on the paradigm of Eisen et al. [38].

**Figure 2:**
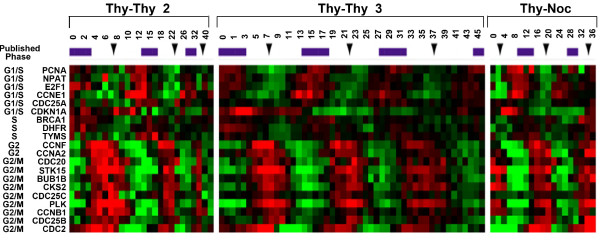
**Gene expression during the HeLa cell cycle**. Each gene corresponds to a row, and the time point (minutes) for each column is denoted. The magnitude of mRNA expression ratio for each gene is represented by different colours. Red indicates mRNA abundance; green indicates a dearth; and gray indicates absence of the data. On the top bar(s), S phase and the time of mitoses (the arrows) are indicated, which was estimated by flow cytometry and BrdU (bromodeoxyuridine) labeling previously.

In the second explored dataset, the genome-wide programming of gene expressions during the cell cycling of a human cancer cell line (HeLa) was characterized using cDNA microarrays. We used part of the microarray dataset obtained from HeLa cells that were synchronized by double thymidine blocking (e.g. *Thy-Thy2 *and *Thy-Thy3*) and thymidine-nocodazole blocking (*Thy-Noc*) [39]. Again, we chose 20 genes for this analysis. Figure 2 displays the temporal profiles for the 20 genes. To learn the regulations, we performed an *n*-fold cross-validation on the *Thy-Thy3 *dataset (47 time points), and then used the datasets, *Thy-Thy2 *(26 time points) and *Thy-Noc *(19 time points), for testing.

In two numerical applications, we considered five units of time delay (*T *= 1, 2, 3, 4, 5). The accuracy of a classifier was estimated through three different ways: by 10-fold cross-validation of *cdc15 *(*Thy-Thy3*), and using the test datasets of *cdc28 *(*Thy-Thy2*) and alpha-factor (*Thy-Noc*). The threshold for the accuracy α, which corresponds to a Type I error of 0.05, was obtained by a permutation approach described later in the Methods section and was used to filter and to define the degrees of certainty for the extracted decision trees: confident, putative or random (see the Methods section for definitions).

### Identification of the time-delayed regulations for yeast cell cycling

**Figure 3:**
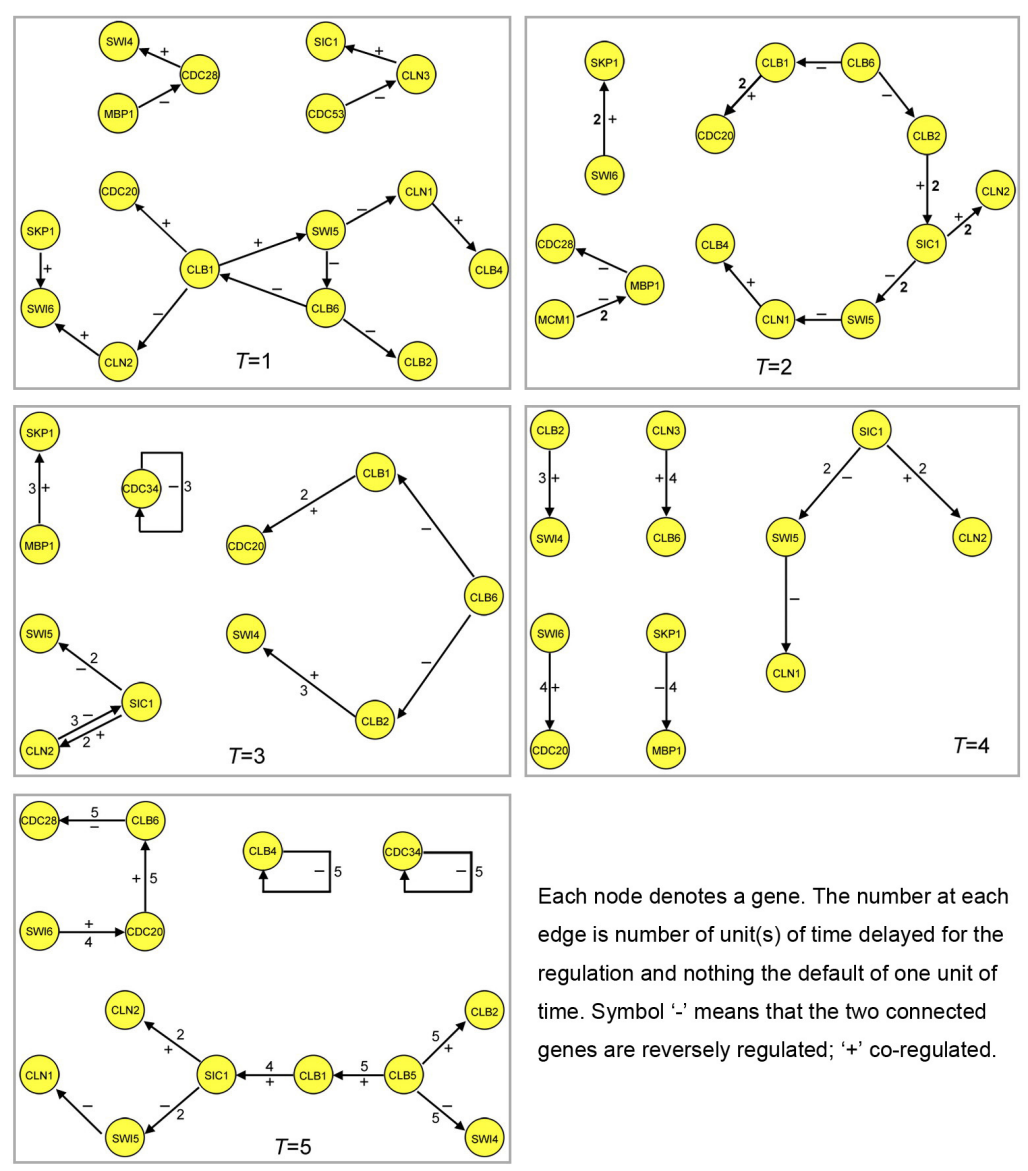
The time-delayed gene regulatory networks for yeast cell cycling.

The accuracies of the classifiers for all the three estimations are given in Additional file 1. The translated gene regulations are given in Table 1. According to the identified regulations, we constructed the graphs of the time-delayed gene regulatory networks (figure 3). To elaborate the longitudinal regulations we defined the following language (see Methods for detail): '+*A*(*t*)' indicates that gene *A *is 'upregulated' at time *t*; '-*A*(*t*)' indicates that gene *A *is 'downregulated' at time *t*; The symbol '=>' represents a directional relationship between genes. For instance, + *A*(*t*) - *B*(*t *- 1) => - *C*(*t *+ 1) means that *C *is 'downregulated' at time *t *+ 1 when *A *is 'upregulated' at time *t *while *B *is 'downregulated' at time *t *- 1.

To verify the biological meanings for the identified genetic relations, we further investigated whether the expression patterns of the genes and the regulation rules are consistent with the phase characteristics of the cell cycle: G1, S, G2, M, M/G1. Yeast cells replicate and divide their genetic material in two distinct but coordinated processes. The S phase is characterized by that the DNA molecule in each chromosome is precisely replicated to form two identical sister chromatids that are held together by cohesions (tethering proteins). During M phase, the cell builds a mitotic spindle, condenses its replicated chromosomes, aligns them on the midplane of the spindle, and then, at anaphase, removes the cohesions and separates sister chromatids to opposite poles of the spindle [22]. The cell divides into two daughter cells shortly after anaphase. There are usually two gaps (G1 and G2) that separate S and M phases. After the M phase, the cell enters the G1 phase and a cell cycle is completed. It has been documented that four classes of elements control the yeast cell-cycle network: cyclins (e.g., CLN1-3, CLB1-2 and CLB4-6); the inhibitors, degraders, and competitors of the cyckin/CDC28 complexes (e.g., SIC1 and CDC20); transcription factors (e.g., MCM1 and SWIs) and checkpoints (the cell size, the DNA replication and damage, and the spindle assembly) [6].

The rules extracted from the confident trees are perfectly consistent with the current knowledge for the cell-cycle gene expression patterns. For examples, we identified the following two rules: +CLB6(*t*) => -CLB1(*t *+ 1) and +CLB6(*t*) => -CLB2(*t *+ 1) (Table 1 and figure 3), which were also found in a previous molecular experiment [40]. Strikingly, a protein-protein networking provided evidence of these relations at protein level [6]. Gene CLB6, which promotes progression of cells into S phase [41], is expressed periodically throughout the cell cycle and is most abundant during late G1 [36,41]. Genes CLB1 and CLB2 both promote cell cycle progression into mitosis [42] and their transcripts accumulate during G2 and M, but their activities are repressed at the end of mitosis [22,36,43,44]. The relation of +CLB1(*t*) => +SWI5(*t *+ 1) (Table 1 and figure 3) was also documented previously [40], where SWI5 encodes a transcription factor that activates transcription of genes expressed at the M/G1 boundary and in G1 phase of the cell cycle [45-47]. SWI5 itself is transcribed in G2 phase [48] and has a maximal expression in G2/M [49,50]. Again, this regulatory rule was identified at protein level previously [6].

Some confident trees and putative trees implicate the same or similar relations. For instances, from confident trees, we identified two relations that regulate genes CLB1 and CLB2: +CLB6(*t*) => -CLB1(*t *+ 1) at *T *= 1, 3; and +CLB6(*t*) =>-CLB2(*t *+ 1) at *T *= 1, 2 (Table 1 and figure 3). The same relations were identified from putative trees (at *T *= 2 for CLB1 and *T *= 3 for CLB2). Many rules extracted from putative trees (Table 1 and figure 3) also have clear biological explanations and evidence. For example, the relation of -SWI5(*t*) => +CLN1(*t *+ 1) was identified previously [40], where CLN1 encodes the yeast cyclins involved in the G1 to S phase transition and the transcription for CLN1 is G1 specific [22,44]. The rule of -SIC1(*t *- 1) => -CLN2(*t *+ 1) is in good agreement with the current knowledge for yeast phase characteristics. The maximum of SIC1 transcriptions are in M/G1, whereas CLN2, which encodes a G1 cyclin, has the peak expression in G1 [51].

The TdGRN modeling is robust to variation of *T*, which is in agreement with the previous result that the yeast cell-cycle network is rather stable against perturbations [6]. We can draw the same conclusion when we change the value of *T*. For instance, when *T *= 1, 2, 4, 5 we identified the same rule, -SWI5(*t*) => +CLN1(*t *+ 1). At *T *= 3, we could also extract this regulation at a lower accuracy. If some regulations were predominating at certain time points, we could still identify the rest at other time points. Take CLN2 as an example. When *T *= 1, we identified +CLB1(*t*) => -CLN2(*t *+ 1) but -SIC1(*t *- 1) => -CLN2(*t *+ 1) was identified at *T *= 2, 3, 4, 5,. We speculate that in the scenario of one unit of time delayed, CLB1 repressed the transcription of CLN2 while the activation of SIC1 to CLN2 was dominative when *T *was larger than one. However, there may be other reasons for a same regulation being identified repeatedly as increasing the range of *T*: (1) it might be related with the high periodicity of the involved genes or with the long-lasting behaviours of the regulation; (2) it might be due to a number of shortcomings that arise with the approach used or for that matter any approach; (3) most of the data generated was not uniformly sampled so although we used only time-points that are equidistant there is no easy way to check if we have data for the right control points when a regulation happens; and (4) a further explanation could be that we see such interactions because the underlying process is not stationary as the cell-cycle has many phases. However this would happen for time-points that are more spread out.

Several genes (CLB5, MCM1, CDC53 and HCT1) did not generate any meaningful rules. There are several possible reasons for the results. One obvious limitation of the microarray technology is that it gives us information about gene regulations only at the level of transcription. Nevertheless, some regulatory interactions between genes may be at protein level, which cannot be revealed directly using miroarray experiments. For example, Li et al. [6] identified several regulatory rules for CLB5 and MCM1 at protein level. Furthermore, mRNA extractions in the *cdc15 *experiment were made every 10 minutes during three cell cycles, which may not be frequent enough to observe all the events.

**Figure 4:**
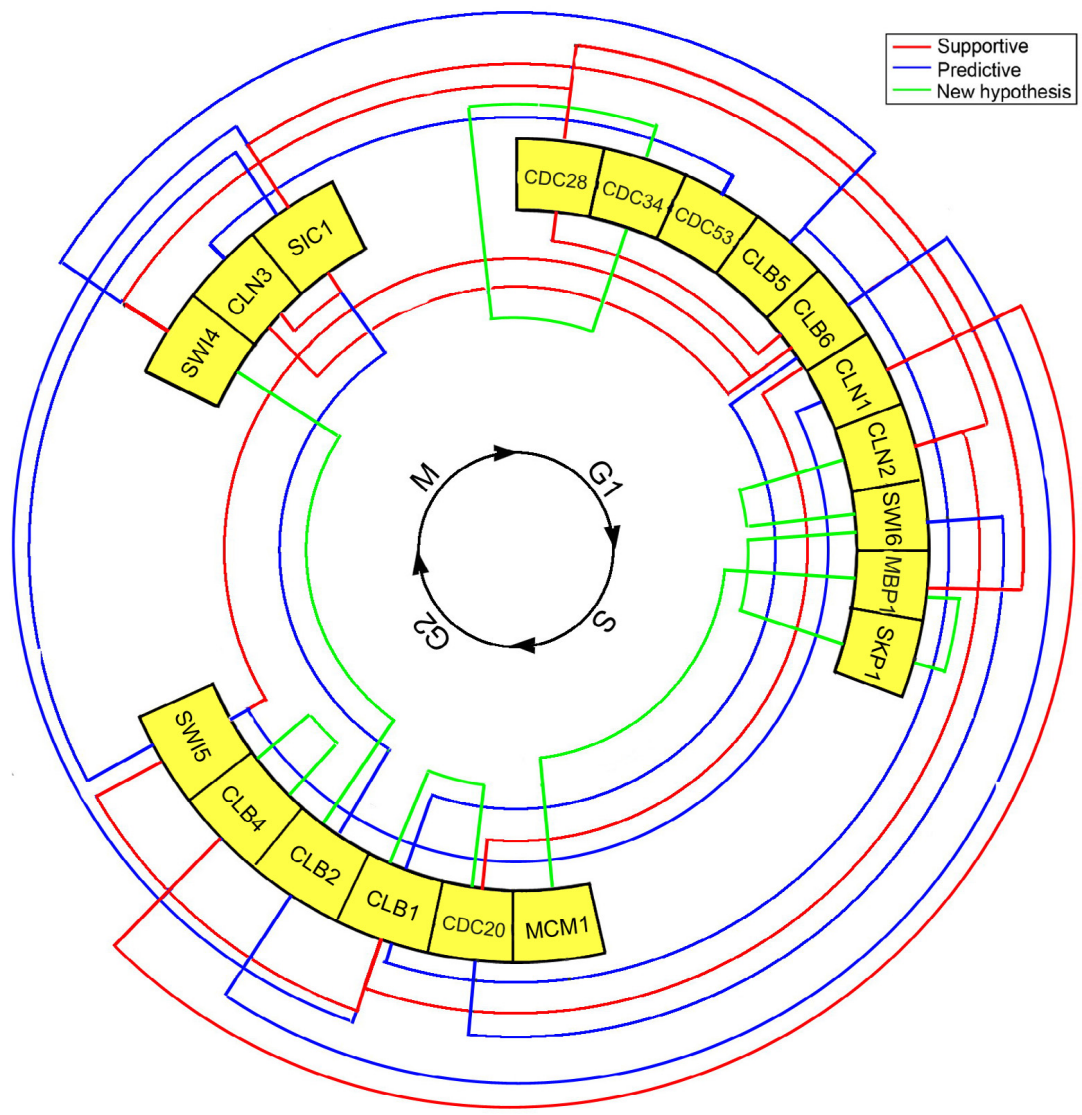
The degree of biological support for yeast cell cycling.

Additional file 2 and Figure 4 give a brief summary from comprehensive biological support analysis of the identified gene regulations for Yeast cell cycling. In total, we identified 32 statistically significant multiple-time-delayed gene regulations for the studied 20 genes involved in *Saccharomyces Cerevisiae *cell cycling. Then, we subject these gene-gene relationships to biological verifications using the knowledge pools of KEGG [52], SGD, and CYGD [53] databases. For this purpose, we defined three categories of biological evidence: supportive if there is explicit and direct experimental evidence demonstrating presence of such a regulatory relationship; predictive if previously documented evidence implies the possibilities of the regulatory interplays between the genes as defined in the multiple-time-delayed gene regulations, but the exact time-delayed mechanism(s) remains to be experimentally verified; and new hypothetical if the biological knowledge for the regulation is totally lacking so far. After performing the comprehensive knowledge searching, we found that 72% of 32 uncovered relations (11 in supportive category and 12 in predictive category) are biologically sounding and documented previously, and the remaining 9 new hypothetical ones lack knowledge so far (see Table S2 in Additional file 2 for detail). However, we might miss some important biological evidence such as documented in other languages, and unpublished results performed by individual labs. The knowledge for most of supportive relationships is mainly derived from and cross-verified by multiple sources (articles and databases). For example, for the mechanism that gene CLB1 inhibits gene CLN2, we found multiple lines of evidence in SGD database and PUBMED articles to support that the G2 cyclins CLB1P, CLB2P, CLB3P, and CLB4P inhibit CLN1 and CLN2 transcription.

### Identification of the time-delayed regulations for human HeLa cell cycling

**Figure 5:**
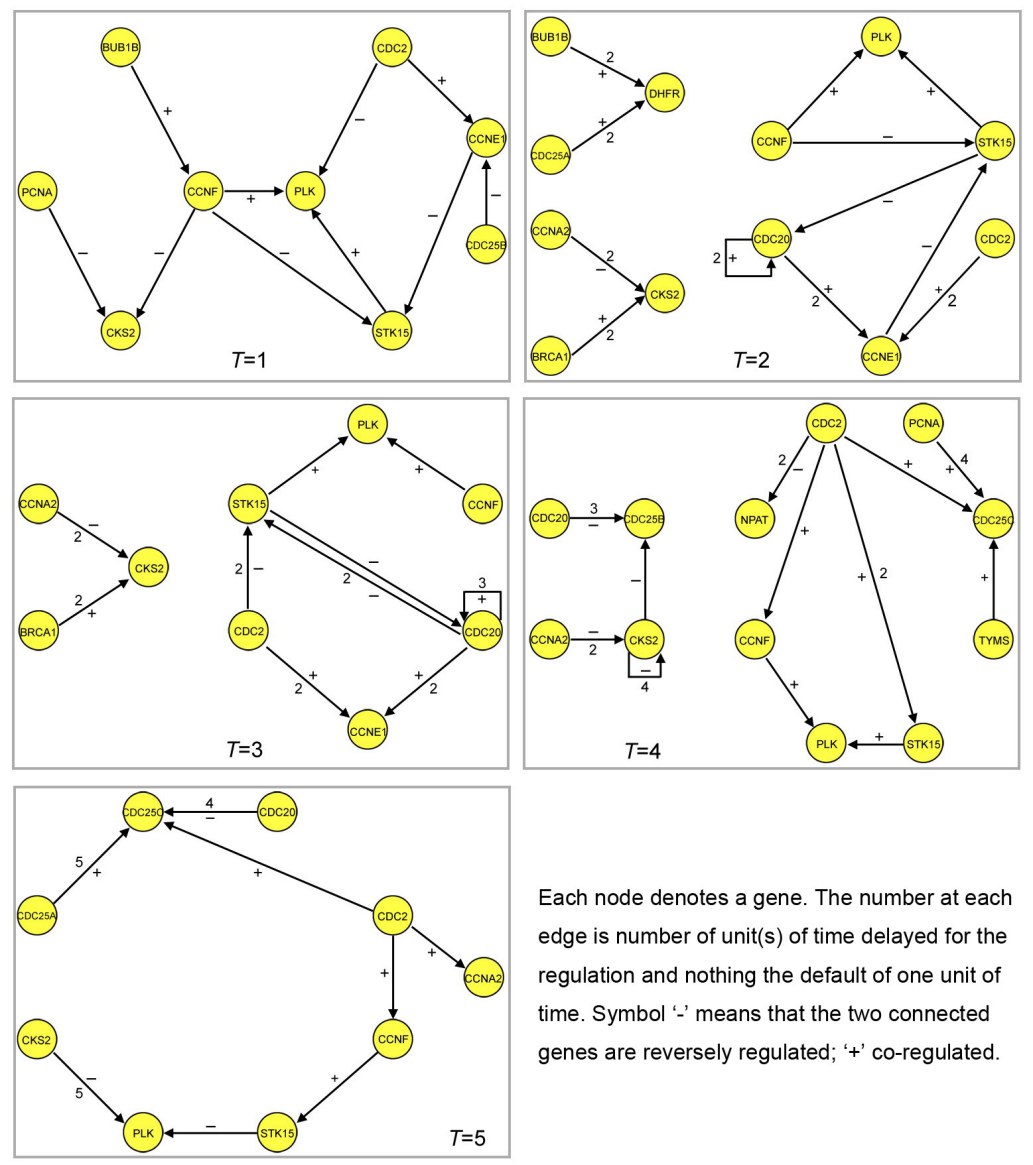
The time-delayed gene regulatory networks for the human HeLa cell cycling.

The human HeLa cell cycling dataset used in this study was part of a large-scale genome-wide program of gene transcriptional profiling during the cell division in a human cancer cell line [39]. The purpose of the previous study was to identify genes periodically expressed in the human cell cycle and to provide a comprehensive catalog of cell cycle regulation genes that can serve as a starting point for functional discovery. Thus, in some sense, our analysis can be considered to be a further work of the previous study — to identify their functional relations between the cell cycle regulation genes. Using spotted cDNA microarrays, containing 22,692 elements representing ~16,322 different human genes or containing 43,198 elements representing ~29,621 genes (estimated by UNIGENE clusters), Whitfield et al. [39] identified a list of the periodically expressed genes based on the estimates of "periodicity score". They found that most of these periodically expressed genes had previously been reported to correlate with the proliferative state of tumors. As the method of periodicity score that Whitfield et al. [39] used in their analysis has conceptually the same or similar basis as the one we proposed here for networking, i.e., to capture the temporal trends of the cell cycle regulated genes, comparison of our results with the previous ones produced many interesting consistencies. Our results for the learnt classifiers' accuracies and the extracted gene regulations are given in Additional file 3 and Table 2, respectively. The derived time-delayed gene regulatory networks for 20 Hela cell cycling genes are shown in Figure 5.

The most striking consistency are our discoveries of the highly confident regulations for STK15 and PLK (see Tables 2 and Figure 5), the top two cell cycle regulated genes with the highest periodicity scores of 58.8 and 56.0, respectively [39]. Both genes had peak expression at the phase G2/M and had been together mapped onto a separate mitotic cluster [39]. Lengauer et al. [54] showed that the two genes have roles in centrosome duplication, whose improper expression is a potential cause of tumor aneuploidy. It is fully logical that we could discover the highly confident gene regulations at all the time spans explored for both genes. It is worth to note that the activity of STK15 was negatively regulated by genes CCNF and CCNE1, whose peak expression was at G1/S and G2, respectively. The temporally sequential nature of the identified regulations might well establish a causal relationship between the regulating genes and the regulated gene. At all the time spans, we found that PLK was regulated by STK15 and/or CCNF. The interactions between the three genes can be seen clearly on the integrated topology as shown in Figure 5.

We also obtained interesting results for gene BRCA1, for which no single putative or confident regulation was identified (Table 2 and Figure 5). Although BRCA1 may be a cell cycle regulated gene based on its periodicity score (5.17), the G1 and/or S phase gene showed heterogeneous expression in tumours, suggesting that the regulation of the gene is more complex than simple restriction of transcription to a particular phase of the cell division cycle [39]. However, the result that no single putative or confident regulation for CCNB1 (cyclin B1) was identified is not expected because based on its periodicity score (24.3) it was considered to be one of representative cell cycle genes [39]. Interestingly, Whitfield et al. [39] also failed to assign this gene by cell cycle phase because CCNB1 fell just outside one of three identified mitotic clusters. A recent study [55] suggests that high expression of cyclin B1 predicts a favourable outcome of patients with follicular lymphoma. We thus conceive that this cell cycle regulated gene may have a role in maintaining normal proliferation of human cells, but is not responsible for the aberrant duplication behaviours in cancer cells

Because of the scope of the paper, we only highlight a few of the identified gene regulations for human HeLa cell cycles. The first groups to be highlighted are the regulations of +CDC2(*t*) +- other genes => +CDC25C(*t+*1) (Table 2 and Figure 5), which were identified from confident trees and/or putative trees. CDC2 (cell division cycle 2) is a member of the Ser/Thr protein kinase family. This protein is a catalytic subunit of the highly conserved protein kinase complex known as M-phase promoting factor (MPF), which is essential for G1/S and G2/M phase transitions of eukaryotic cell cycle. CDC25C (cell division cycle 25C) is a tyrosine phosphatase and belongs to the CDC25 phosphatase family. It plays a key role in the regulation of cell division. Cyclin B-Cdc2 can phosphorylate and activate CDC25C, forming a positive feedback loop that contributes to the abrupt transition from G2 into M phase [56,57]. These previous facts have convincingly validated our networking results.

The second highlight is gene E2F1, which was identified to be a regulator for PCNA and CDC2: +E2F1(*t *- 1) +- other genes => +PCNA(*t *+ 1) and -E2F1(*t *- 4) => -CDC2(*t *+ 1) (Table 2 and Figure 5). Strikingly, both regulations were demonstrated using Northern blots assays [58]. Many of genes expressed at G1/S and S phase are known E2F target. A study of the cell cycle and the E2F transcription factors in mouse embryo fibroblast, using microarray, identified both G1/S and S phase genes, as well as genes expressed at G2 and M phase, as targets of E2F [59]. The protein encoded by E2F1 is a member of the E2F family of transcription factors. The E2F family plays a crucial role in control of the cell cycling. The protein encoded by PCNA was found in the nucleus and is a cofactor of DNA polymerase delta. The encoded protein acts as a homotrimer and helps increase the processivity of leading strand synthesis during DNA replication. Many of the genes that encodes S-phase-acting proteins, including DNA polymerase alpha, thymidylate synthase, proliferating cell nuclear antigen, and ribonucleotide reductase, are in fact induced by E2F1. In addition to the S-phase genes, several genes that play regulatory roles in cell cycle progression, such as the cdc2, cyclin A, and B-myb genes, are also induced by E2F1 [58].

Strong experimental evidence can also be established for the regulation of CDC20: -STK15(*t*) +CDC20(*t *- 1) => +CDC20(*t *+ 1) (Table 2 and Figure 5). CDC20/fizzy family proteins are involved in activation of the anaphase-promoting complex/cyclosome, which catalyzes the ubiquitin-dependent proteolysis of cell cycle regulatory proteins such as anaphase inhibitors and mitotic cyclins, leading to chromosome segregation and exit from mitosis. Aurora2/Aik (STK15), a member of the aurora/Ip11 family of kinases, was implicated previously in the pathways regulating chromosome segregation. In HeLa cells, CDC20 is associated with the kinase aurora2/Aik because this enzyme could regulate the function of CDC20 that often act as a targeting subunit for aurora2/Aik [60]. The finally highlighted gene regulation is +CDC2(*t*) => +CCNA2(*t *+ 1) (Table 2 and Figure 5), where CCNA2 (cyclin A2) belongs to the highly conserved cyclin family, whose members are characterized by a dramatic periodicity in protein abundance through the cell cycles. Yam et al. [61] presented evidence from in vitro and in vivo assay systems that the degradation of human cyclin A can be inhibited by kinase-inactive mutants of CDC2.

Additional file 4 gives a brief summary from comprehensive biological support analysis of the identified gene regulations for 20 Hela cell cycling genes. Totally, we extracted 58 time-delayed gene regulations. By considering 24 overlapping ones, there are 44 unique gene-gene relationships. The detailed explanation for each time-delayed regulation is given in Table S4 in the additional file. We annotated our results with PUBMED, Entrez Gene, BIND [62] and KEGG databases. Based on the degree of biological support, 18 time-delayed regulations are supportive, 10 predictive and 16 new hypothetical. Therefore, 64% of the uncovered relations (18 supportive and 10 predictive) are biologically sounding and documented previously.

Overall, in most cases, the extracted rules for both yeast and human HeLa cell cycling confirm the current knowledge about their gene regulations, while hitherto a few newly discovered ones can be treated as the novel hypotheses to be verified and may define novel genetic pathways.

## Discussion

In this paper, we have introduced the TdGRN method (Time-delayed Gene Regulatory Network) to address genetic dependencies with the time frames over more than one unit of time. We used a decision tree to discover the time-delayed regulations between the underlying genes. The main advantages of the proposed method are as follows. First, it can be efficiently used to unravel the gene regulations delayed *T *units of time apart without priori discretisation of the continuous gene expression data to circumvent the information loss. Essentially, it is free from the problem associated with definition of the number of regulating genes, *k*, which is often arbitrarily determined. Second, the regulations are extracted in parallel with construction of decision trees. It thus enjoys the merit of easy interpretation. Third, time series data are not the only type of data to which our method is applicable. It is straightforward to use the novel algorithm to explore various cases where potential dependencies between different experimental units might occur, for example, to identify the regulations related to the development, aging, and the progressive pathogenesis of a complex disease at molecular levels.

However, several issues for the proposed method are warranted further investigations. Although the empirical threshold for prediction accuracy, determined by permutations, provides a robust measure of statistical significance for a regulation rule, directly working on a large feature gene set of thousands of genes can be computationally high demanding. Nevertheless, use of our newly proposed ensemble approach for dimensional reduction and for mining the target-relevant genes can efficiently solve this issue [63]. Alternatively, before reversely engineering the underlying gene networks, one may consider using "periodicity score" method to identify periodically expressed genes [36,39]. Our proposed method has some analogies to the "periodicity score" method. The latter was mainly used to capture the temporal characteristics of an individual gene by modelling the time series trends of the genes using a known function such as a Fourier transform although the gene expression profiles of many cell cycle genes do not precisely match sine and cosine curves. In fact, this kind of data reduction approach is similar to the use of summary measures (mean, slope and principal components) of multiple longitudinal data in repeated measure analysis [64]. Recently, hidden Markov models have been increasingly applied to analysis of temporal gene expression data such as for yeast cell cycling [32,65] and can be considered being used for both identification of periodically expressed genes and gene networking because additional time series correlations within and between cell cycling genes can be taken into properly.

Identification of cell cycling feature genes provides a comprehensive list of cell cycle regulation genes for exploring the more involved gene-gene interactions. In some sense, to unravel their functional relations between the cell cycle regulation genes can be considered to be a continual work and is a focus in modern functional genomics. For the purpose of demonstration, we applied to 20 genes for each of the two publicly available datasets. Nevertheless, larger gene sets would not impose a difficulty in application of the proposed algorithm as tree models used in this study has been demonstrated to be robust to a dimension curse [63]. Despite this fact, directly networking genes using the raw expression data of thousands of genes is not a recommended strategy as this analysis strategy may introduce noises in the gene networks and significantly increases the computing load. Therefore, an ideal way is to extract the optimal regulating (and the regulated) gene subset beforehand, e.g. using a robust global search algorithm such as a hybrid between genetic algorithm and support vector machines that we developed recently [66]. In the proposed method, we built gene regulations for each target gene separately. To deduce larger gene-interaction networks, one can combine the results for all the target genes to construct larger networks of gene inter-relationship by connecting genes by directed edges according to the identified regulations, as done in Figures 3 and 5. It should be noted that a globally optimal gene network for a biological mechanism may not be accomplished using a single dataset (or experiment). However, the proposed TdGRN model can easily accommodate new data acquired further to accumulate our knowledge for the gene-gene interplays gradually till the fully elucidated genetic architecture is obtained.

Regarding which, a model-based or model-free approach, is more efficient for dissecting the dynamic mechanisms of the longitudinal gene-gene regulations, the debates will perhaps continue. In our own opinions, instead treating the two types of approaches as competitors, integrating their respective merits is expected to be more helpful in probing the mysteries of the underlying gene networking mechanisms. Based on theories of statistical inference, it can be proved that the elegant model-based dynamic models with a solid distribution-theory basis are mathematically more sounding and more powerful than model-free approaches without relying on the support. However, before a powerful model-based approach is applied to a practical forum, one has to assure that the underlying complexities can be depicted fairly accurately by the model, which would remain to be a challenging issue prior to accumulation of sufficient knowledge about the biological system or one can consider using a less strict model-free approach as a first-cut networking mining tool. In our recent study for exploring modes of gene-gene relations for Hela cell cycling (brief results are given in Additional file 5), we found that the gene-gene relations almost equal-likely follow three common modes (parallel, time-shifted and inverted), identified by a local alignment algorithm [67]. We observed that the relationships for the gene pairs that are expressed in parallel or time-shifted manner are only obvious in the same or the neighbouring cell cycle phases, while for the gene pairs that are of the inverted relationship, their transcriptional activities span at least one phase apart. These data have two important implications for computational gene networking. First, the traditional static networking approaches (i.e. without modelling the time-delayed effects) are limited for analyzing such time-series data. Second, the mixture of three relation modes would impose additional difficulties in precisely mathematical modelling. Consequently, a robust time-delayed gene networking (against both intrinsic noise in the microarray data and inherent biological complexities) is highly demanding.

Our proposed TdGRN can be rendered a well-conceived model-free approach that attempts to learn the underlying regulatory rules without relying on any model assumptions (e.g. the network architecture, the number of regulating genes, and so on). Although we have seen several published methods for exploring the time trends of transcriptional activities on per gene basis, to our knowledge, we are among the pioneering groups to formalize a systematic model-free approach to explore the dynamic properties and behaviours of multiple-time-delayed gene regulations. Because of the very nature of the method, we would rather consider TdGRN as a tactful analysis strategy whose major goal is to transform massive biological data into a simple mechanistic understanding. In addition, we have performed a comprehensive biological support analysis for the identified time-delayed regulations by matching various knowledge databases such as Entrez Gene, PUBMED, KEGG, BIND, SGD and CYGD. The knowledge mining has demonstrated that most of the gene regulations identified by TdGRN enjoy good biological evidence support. We found that 72% of 32 uncovered regulations for Yeast and 64% of 44 uncovered regulations for Hela cells are biologically sounding and evidence-based. These results have well established the robust bioinformatics toolbox as a promising and feasible computational approach to generating a working blueprint for mapping the dynamic mechanisms of time-delayed gene regulations.

The exponential increases of the amount of massive time-series data have offered rich opportunities to uncover causal gene-gene or gene-phenotype relationships. Time series data structure offers a necessary (but not sufficient) condition – time lag to infer a cause-effect relationship [30]. Despite its fundamental importance in modern biomedicine, to uncover cause-effect relationships is yet a very challenging topic for computational biologists. From a methodological view, many computational tools are "correlation-" or "association-" based. Strictly speaking, such models are not able to reveal causal gene-gene or gene-phenotype relationships. The identified relationships are of bi-directions or no direction at all. The typical algorithms are various clustering approaches and distance measures that are motivated by the hypothesis that genes with similar expression profiles are likely to be co-regulated. Thus, a high gene-gene correlation (or anti-correlation) measured by these approaches can be due to the fact that (1) gene *A *regulates gene *B*; or (2) gene *B *regulates gene *A *or (3) genes *A *and *B *are being co-regulated by a third gene *C*; or (4) accidental. The proposed TdGRN method attempts to identify the relationship between the genes whose activities can be delayed by multiple time points. A gene at time *t *+ 1 is potentially regulated (triggered) by the genes at previous (not later) time points {*t*, *t *- 1, …, *t *- (*T *- 1)}. This longitudinal configuration for the regulated gene and the regulating genes meets the necessary condition (time lag) for a cause-effect relationship to occur. The decision-tree learning core for TdGRN makes multiple-layer decisions at the recursive partitions to capture both the individual effects of a regulating gene *g*_*A *_and its synergic effects with other attributed genes that are imposed on the activity states of the target gene *g*_*i*_. Then, we employed very strict accuracy criteria to remove any accidental "cause-effect" relationship. Thus, we believe that the time-delayed gene-gene relationships identified by TdGRN are more likely to be causal. Nevertheless, it should be cautioned that the proposed model-free approach cannot distinguish well between a causal gene-gene regulation and the scenarios where genes A and B are being co-regulated by a third gene C, which can be further elaborated using a suitable model-based approach or a well-designed molecular experiment.

## Conclusion

In summary, we have described a novel model-free approach for reconstructing the time-delayed gene regulatory networks. We have applied the proposed method to yeast cell cycling and human HeLa cell cycling and have discovered most of the underlying time-delayed regulations that are supported by multiple lines of experimental evidence and that are remarkably consistent with the current knowledge on phase characteristics for the cell cyclings. The regulations extracted from confident trees are perfectly consistent with the current knowledge for the cell-cycle gene expression patterns. This novel approach can be efficiently used to unravel the gene regulations delayed *T *units of time apart without priori discretisation of the continuous gene expression data and is robust to variation of *T*. Furthermore, the regulations are extracted in parallel with construction of decision trees. It thus enjoys the merit of easy interpretation.

## Methods

### Constructing the time-delayed gene expression profiles (*TdE*)

Gene expression profile can be represented as an *n *× *m *matrix, *E *= (*e*_*ij*_), where each row represents a gene and each column represents expression values measured under different experimental conditions, or different physiological and developmental stages, or the data obtained by monitoring the expression levels of a gene at different time points involved in a biological process (e.g., cell cycles). The element *e*_*ij *_in row *i *and column *j *of the *E *matrix denotes the expression level of gene *g*_*i *_in the *j *th measurement. In the TdGRN model, a gene at time *t *+ 1 is potentially regulated by the genes at previous time points (say, time points *t*, *t *- 1, …, *t *- (*T *- 1)), where *T *is the maximal time span explored). We thus reshaped the *E *matrix into *TdE *to elucidate the time-delayed effects.

The method to construct *TdE *is given as follows. Assume that the gene expression profile *E *is an *n *× *m *matrix. *TdE *will then be an (*m *- *T*) × (*n *× *T*) matrix, where each *T*-columns block in the *n *× *T *columns represents the activities of each of the *n *(regulating) genes at time points *t*, *t *- 1, …, *t-*(*T *- 1) and each row is therefore an (*n *× *T*)-dimension vector. As the value of *t *changes from *T *to *m *- 1, the time window moves from the first time point to the *m *- *T *time point, it produces *m *- *T *such vectors or called *m *- *T *samples. Next, we set up the corresponding phenotype (label) for each sample, which was determined by the states of the target (regulated) gene (*g*_*i*_). The completed data for the time-delayed gene expression profiles for the target gene were denoted by *D*_*i *_= (*TdE*, *C*_*i*_), where *C*_*i *_is a column vector of states for gene *g*_*i*_. In our method, we assumed that the transcription machinery of gene *g*_*i *_can be in a finite number of different states, and that the expression of the gene is determined by its state. The flexibility of the approach is that we can explore different interpretations of states. For simplicity, we only reported the scenario for two states, i.e., 'upregulated' or 'downregulated' here. More precisely, we defined a state function σ_*i *_for gene *g*_*i *_such that given its real expression value *e*_*ik *_(at time point *k *= *T *+ 1, *T *+ 2, ..., *m*) it returns a value (*C*_*ik*_) from a discrete domain. Let us assume that σ_*i *_is a function that returns '1' if gene *g*_*i *_is 'downregulated' and '2' if it is 'upregulated'. As the expression values in the analyzed datasets were the log ratios, we therefore chose the zero as the threshold to distinguish the two different states. Thus, in this particular case, σ_*i *_(*e*_*ik*_) ∈ {1, 2}. Given the expression value *e*_*ik *_of gene *g*_*i*_, and the σ_*i *_function, we defined the state of the target gene as:

Cik=σi(eik)={2 (upregulated)if eik>01 (downregulated)otherwise
 MathType@MTEF@5@5@+=feaafiart1ev1aaatCvAUfKttLearuWrP9MDH5MBPbIqV92AaeXatLxBI9gBaebbnrfifHhDYfgasaacH8akY=wiFfYdH8Gipec8Eeeu0xXdbba9frFj0=OqFfea0dXdd9vqai=hGuQ8kuc9pgc9s8qqaq=dirpe0xb9q8qiLsFr0=vr0=vr0dc8meaabaqaciaacaGaaeqabaqabeGadaaakeaacqWGdbWqdaWgaaWcbaGaemyAaKMaem4AaSgabeaakiabg2da9iabeo8aZnaaBaaaleaacqWGPbqAaeqaaOGaeiikaGIaemyzau2aaSbaaSqaaiabdMgaPjabdUgaRbqabaGccqGGPaqkcqGH9aqpdaGabaqaauaabaqaciaaaeaacqaIYaGmcaaMc8UaeiikaGIaemyDauNaemiCaaNaemOCaiNaemyzauMaem4zaCMaemyDauNaemiBaWMaemyyaeMaemiDaqNaemyzauMaemizaqMaeiykaKcabaGaemyAaKMaemOzayMaaGPaVlabdwgaLnaaBaaaleaacqWGPbqAcqWGRbWAaeqaaOGaeyOpa4JaeGimaadabaGaeGymaeJaaGPaVlabcIcaOiabdsgaKjabd+gaVjabdEha3jabd6gaUjabdkhaYjabdwgaLjabdEgaNjabdwha1jabdYgaSjabdggaHjabdsha0jabdwgaLjabdsgaKjabcMcaPaqaaiabd+gaVjabdsha0jabdIgaOjabdwgaLjabdkhaYjabdEha3jabdMgaPjabdohaZjabdwgaLbaaaiaawUhaaaaa@7D84@

The *D*_*i *_= (*TdE*, *C*_*i*_) matrix for the target gene *g*_*i *_is given in Table 3.

### TdGRN method

The goal for the TdGRN modelling was to unravel the time-delayed gene regulations. The basic idea is that for the target gene *g*_*i *_we seek for the interacting attribute gene *g*_*A *_that regulates the expression of gene *g*_*i*_. The inputs for TdGRN are *D*_*i *_and *T *(the maximal time span explored), while the outputs are the relations between the regulating genes and the target regulated gene. Thus, in some sense, this modelling can be conceived to be the search of the relevant biological decision rules. We defined a classifier, *I*, as a function that maps a sample of *TdE *to a discrete value. In this study, we applied a decision tree as the learner [63]. Identification of biological regulatory rules parallels with the construction of an inverse tree, starting from root and ending with leaves (terminal nodes) or till a stopping rule for tree growth was satisfied.

### Evaluating the learnt regulations

In order to identify significant and meaningful regulations between genes, we filtered out the adequate decision trees first. For this purpose, we defined three degrees of certainty for a tree. If a decision tree achieved accuracies higher than a specified threshold (α) in all evaluation datasets (either different experimental datasets or a cross-validation permutated replicate), we deemed it to be a confident tree. If a decision tree achieved accuracies higher than a specified threshold (α) in some evaluation datasets but not in others, we conceived it of being a putative tree. If the accuracies of a decision tree in all evaluations were lower than α, we defined it to be a random tree. In order to minimize the risk of extracting some false positives occurring by chance, we discarded those random trees. We used permutations to identify the threshold at the Type I error of 0.05. The detailed procedures for permutation are given in the next section.

**Figure 6:**
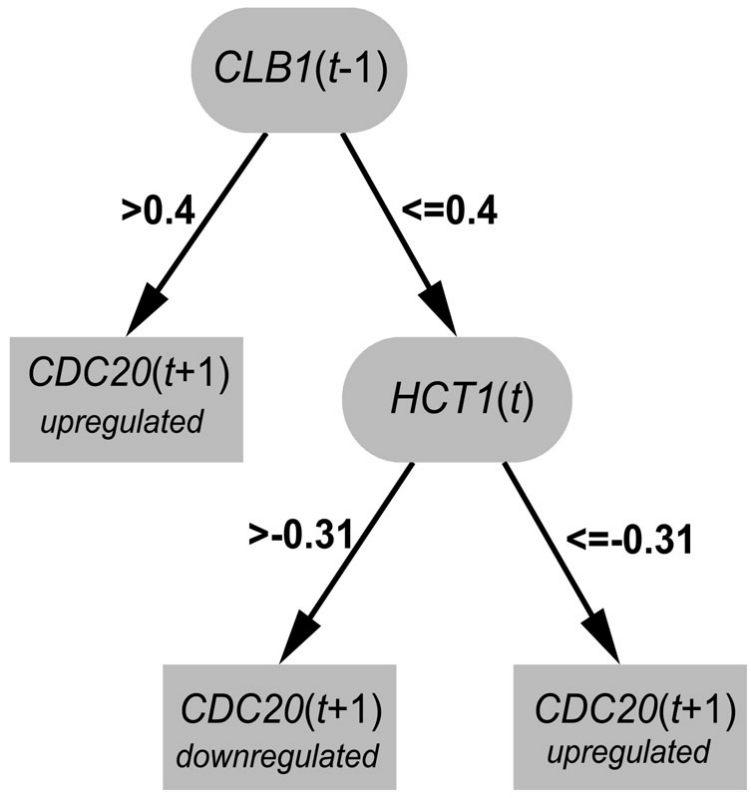
Decision tree for gene CDC20 as an example for extraction of regulatory rules.

In a decision tree, each path from root to a leaf defines a regulatory rule. The rule +CLB1(*t *- 1) => +CDC20(*t *+ 1) in Figure 6 is straightforward and can be interpreted in terms of 'upregulated' or 'downregulated' because there is only one branch in this direction ('CLB1(*t *-1) > 0.4' implicates that expression of CLB1 at time *t *- 1 is absolutely upregulated). Therefore, we can infer that the upregulation of CDC20 at time *t *+ 1 is resulted from the rising of CLB1's expression level at time *t *- 1. However, the other branches are more difficult to be interpreted. The fact that CLB1(*t *- 1) ≤ 0.4 does not unambiguously imply regulations of 'upregulated' or 'downregulated' (the trend that the value changes from 0 to 0.4 is 'upregulated' or *vice versa *'downregulated'). Nevertheless, this branch does not mean that the genes are irrelevant and more explicit rules are required to clarify the ambiguity.

### Identifying the accuracy threshold (α) by permutations

We used permutations to identify the accuracy threshold (α) corresponding to a specified Type I error rate for the null hypothesis of no such a rule(s). First, we fixed the percentage of samples in every class (i.e., a state of the target gene). We then randomly shuffled the phenotypes (labels for the expression states of the target gene) of the samples contained in *D*_*i *_= (*TdE*, *C*_*i*_). The permuted *D*_*i *_is thus a randomized replicate (*ranD*_*i*_), on which we built a recursive partition tree. We repeated the same permutation procedure 500 times and defined the 95% quantile as the empirical threshold that corresponds to the Type I error of 0.05. We determined the threshold for every delayed time point for each gene separately (the data are given in Additional files 6 and 7).

### Computational algorithms

**Figure 7:**
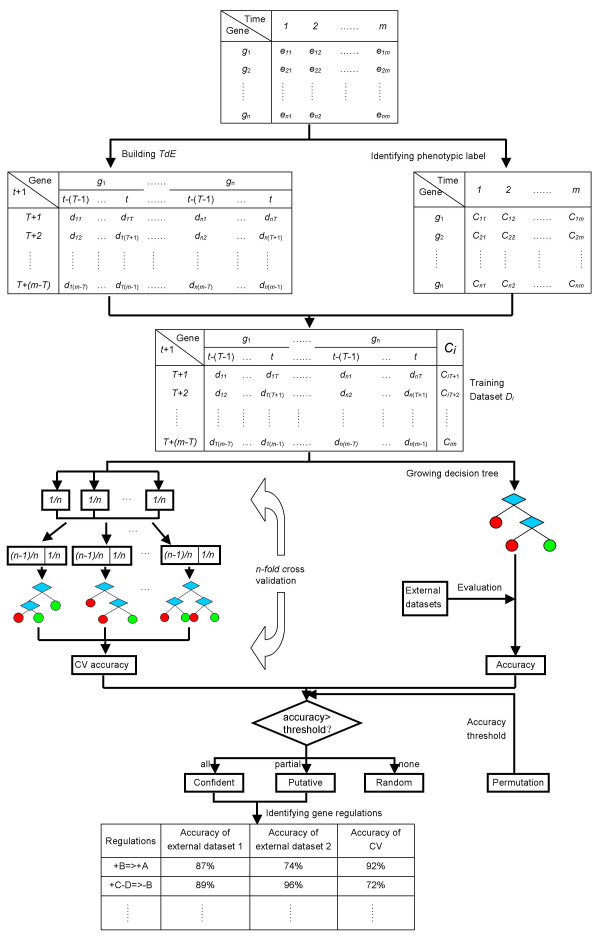
The graphic algorithm flow of the TdGRN method.

The numeric algorithm for the TdGRN model, organized step-by-step, is described below and graphically depicted by Figure 7. All the subroutines have been realized on the MATLAB platform. The corresponding programming codes are available upon written request to the authors. The topological graphs of the time-delayed gene regulatory networks were drawn with the Graph Editor Toolkit of the Tom Sawyer Software Series [68].

**Step 1: **Building the time-delayed gene expression profiles (*TdE*):

1. Define a time window size of *T *and set the target gene at the time *T *+ 1. Organize expression data for each of *n *genes in the order of time points 1, 2, …, *T*. Therefore, for *n *genes, it is an *n *× *T*-dimension vector. We call this vector the first sample of *TdE*.

2. Shift the time window to next time point (and now the target gene is at the time *T*+2). Again, organize expression data for each of *n *genes in the order of time points 2, 3, …, *T *+ 1. We called the *n *× *T*-dimension vector for *n *genes the second sample of *TdE*.

3. The remaining sample vectors can be built similarly until the right window margin reaches the time point of *m*-1 (and now the target gene at the last time point explored). The last sample vector consists of the expression data for *n *genes at time points *m *- *T*, *m*-*T *+ 1, …, *m *- 1. Thus, there are totally *m *- *T *sample vectors (or samples for short) in matrix *TdE*.

**Step 2: **Assigning a phenotypic label for each sample and building up the training dataset *D*_*i *_(for the target gene *g*_*i*_):

1. If the expression level (the log ratio value) of the target gene at a time point (*T *+ 1, ..., *m*) is bigger than zero (i.e., upregulated), the phenotypic label for the corresponding sample is '2'. Otherwise (i.e., downregulated), it is assigned with the label '1'.

2. The labels for *m *- *T *samples are organized with an *m *- *T*-dimension column vector, called phenotypic or state vector. The resulting matrix *D*_*i*_, obtained by merging from sample vectors and phenotypic vector, has dimensions of (*m *- *T*) × (*n *× *T *+ 1) and is used as the basic analysis unit for discovery of multiple time-delayed regulatory networks (for a target gene).

**Step 3: **Identifying time-delayed regulatory rules by growing a decision tree on *D*_*i*_

1. As in the conventional usages of machine learning, each row and column in *TdE *are termed a sample and a feature vector, respectively. That is, there are totally *m *- *T *samples and *n *× *T *features (columns).

2. At an internal node or at the root node of a tree, perform a recursive partition (for detail, see [63]). In brief, at tree node *r*_*i*_(*i *= 0, 1, …), rank in descending order the values for each feature of *TdE*, say *d*_*kl *_(*k *= *T *+ 1, *T *+ 2, …, *T *+ (*m *- *T*)) for the expressions of the feature *d*_*l *_(*l *= 1, …, *n *× *T*) (i.e., a gene at a specific delayed time point). For feature *d*_*l*_, its corresponding partition cutoff was determined by the midpoints of two-ordered values, *b*_*kl *_= *d*_*kl *_+ (*d*_*k *+ 1, *l *_- *d*_*kl*_)/2. Determined by its *d*_*l *_value, a sample was mapped into *w *(here, *w *= 2) discrete values (i.e., assigning a trained state for the target gene). Then, we repeated the same procedures and computed an information gain for each (gene) feature to identify the best at the node that can achieve the maximum of the information gain. The (gene) feature having the biggest information gain was considered to be the best attribute to splitting. The information gain (*Gain *(*C*_*i*_, *g*_*A*_)) of the attribute feature *g*_*A *_(i.e., a gene at a previous time point) to the state set (*C*_*i*_) of the target gene *g*_*i *_was defined as:

Gain(Ci,gA)=Entropy(Ci)−∑v∈Values(gA)|Sv||Ci|Entropy(Sv)
 MathType@MTEF@5@5@+=feaafiart1ev1aaatCvAUfKttLearuWrP9MDH5MBPbIqV92AaeXatLxBI9gBaebbnrfifHhDYfgasaacH8akY=wiFfYdH8Gipec8Eeeu0xXdbba9frFj0=OqFfea0dXdd9vqai=hGuQ8kuc9pgc9s8qqaq=dirpe0xb9q8qiLsFr0=vr0=vr0dc8meaabaqaciaacaGaaeqabaqabeGadaaakeaacqWGhbWrcqWGHbqycqWGPbqAcqWGUbGBcqGGOaakcqWGdbWqdaWgaaWcbaGaemyAaKgabeaakiabcYcaSiabdEgaNnaaBaaaleaacqWGbbqqaeqaaOGaeiykaKIaeyypa0JaemyrauKaemOBa4MaemiDaqNaemOCaiNaem4Ba8MaemiCaaNaemyEaKNaeiikaGIaem4qam0aaSbaaSqaaiabdMgaPbqabaGccqGGPaqkcqGHsisldaaeqbqaamaalaaabaWaaqWaaeaacqWGtbWudaWgaaWcbaGaemODayhabeaaaOGaay5bSlaawIa7aaqaamaaemaabaGaem4qam0aaSbaaSqaaiabdMgaPbqabaaakiaawEa7caGLiWoaaaaaleaacqWG2bGDcqGHiiIZcqWGwbGvcqWGHbqycqWGSbaBcqWG1bqDcqWGLbqzcqWGZbWCcqGGOaakcqWGNbWzdaWgaaadbaGaemyqaeeabeaaliabcMcaPaqab0GaeyyeIuoakiabdweafjabd6gaUjabdsha0jabdkhaYjabd+gaVjabdchaWjabdMha5jabcIcaOiabdofatnaaBaaaleaacqWG2bGDaeqaaOGaeiykaKcaaa@7501@

where, Entropy(Ci)=∑h=1H−phlog2ph
 MathType@MTEF@5@5@+=feaafiart1ev1aaatCvAUfKttLearuWrP9MDH5MBPbIqV92AaeXatLxBI9gBaebbnrfifHhDYfgasaacH8akY=wiFfYdH8Gipec8Eeeu0xXdbba9frFj0=OqFfea0dXdd9vqai=hGuQ8kuc9pgc9s8qqaq=dirpe0xb9q8qiLsFr0=vr0=vr0dc8meaabaqaciaacaGaaeqabaqabeGadaaakeaacqWGfbqrcqWGUbGBcqWG0baDcqWGYbGCcqWGVbWBcqWGWbaCcqWG5bqEcqGGOaakcqWGdbWqdaWgaaWcbaGaemyAaKgabeaakiabcMcaPiabg2da9maaqahabaGaeyOeI0IaemiCaa3aaSbaaSqaaiabdIgaObqabaacbiGccqWFSbaBcqWFVbWBcqWFNbWzdaWgaaWcbaGaeGOmaidabeaakiabdchaWnaaBaaaleaacqWGObaAaeqaaaqaaiabdIgaOjabg2da9iabigdaXaqaaiabdIeaibqdcqGHris5aaaa@4E5D@ and *Values *(*g*_*A*_) include all *w *discrete values of gene *g*_*A *_and *S*_*v *_is a sample subset consisting of the samples whose discrete values are *v *(*v *∈ *Values *(*g*_*A*_)). |*C*_*i*_| and |*S*_*v*_| are the norms for the sets *C*_*i *_and *S*_*v*_, respectively. *p*_*h *_is the proportion of the gene states which are *h *∈ σ_*i *_in *C*_*i*_. In this step, we identified not only the best regulatory gene but also its regulating time.

Next, we used the best gene to bifurcate node *r *into two child nodes, *r*_1 _and *r*_2_, on which we repeated the binary partition analysis until a stopping rule for tree growth was satisfied (i.e. a terminal node contains observations from only one class or has the maximum allowed instances at a node). A decision tree may contain multiple regulatory rules defined by a path from the root node to a terminal node. We thus extracted a path(s) that can unambiguously implicate a gene regulation and abandoned the remaining paths.

**Step 4: **For each grown tree, we used *n*-fold cross validation or external independent datasets to evaluate its accuracy for predicting the activity state of the target gene and to define its degree of certainty as a regulatory rule.

**Step 5: **A random permutation procedure was implemented independently for each of evaluation datasets to provide an accuracy threshold at a specified Type I error rate (e.g., 0.05) that can be a statistical measure for evaluation of a putative decision tree, identified in **Step 4**.

**Step 6: **For the target gene *g*_*i*_, its multiple-time delayed regulations were discovered using the procedures described in **Steps 1–5**. We repeated the same procedures for each of the target genes whose regulations to be sought for.

**Step 7: **Based on the gene regulations extracted for the target genes, we construct the larger gene regulation networks according to different time-delayed frames. We combine the data for all the target genes to construct larger networks of gene inter-relationship by connecting genes by directed edges.

## List of abbreviations used

Time-delayed Gene Regulatory Network (TdGRN), Time-delayed gene expression profiles (*TdE*), Cyclin-dependent kinase (CDK), Thymidine (*Thy*), Nocodazole (*Noc*).

## Authors' contributions

This study was undertaken by a collaborative team of several institutes as indicated. XL, SR and JW conceived of the proposal of the study, conducted the study and drafted the manuscript. The remaining authors participated in writing the computing codes and applied the data mining strategy to the field datasets. All authors participated in reading, approving and revising the manuscript.

## Supplementary Material

Additional File 1 The accuracy (%) of each gene's classifiers in all of the three estimations (cdc15, cdc28 and α-factor) at each delayed time point (T). Click here for file

Additional File 2 The biological support of the gene regulations for Yeast cell cycling. Click here for file

Additional File 3 The accuracy (%) of each gene's classifiers in all of the three estimations (Thy-Thy2, Thy-Noc and Thy-Thy3) at each delayed time point (T). Click here for file

Additional File 4 The biological support of the gene regulations for human HeLa cell cycling. Click here for file

Additional File 5 Modes of the gene-gene relationships for Hela cell cycling. Click here for file

Additional File 6 The empirical accuracy thresholds (α) for each yeast gene at each delayed time point (T) in three estimations (cdc15, cdc28 and α-factor), corresponding to a point-wise Type I error of 0.05. Click here for file

Additional File 7 The empirical accuracy thresholds (α) for each human cancer gene at each delayed time point (T) in three estimations (Thy-Thy2, Thy-Noc and Thy-Thy3), corresponding to a point-wise Type I error of 0.05. Click here for file

## Figures and Tables

**Table 1:** List of the identified time-delayed gene regulations for yeast cell cycling. In the bold style are the rules extracted from confident trees and in the regular style are the rules extracted from putative trees.

Gene name	Peak phase	Time Delayed				
		
		One-unit time	Two-unit time	Three-unit time	Four-unit time	Five-unit time
CLN1	G1	+SWI5(*t*) => -CLN1(*t*+1)	-SWI5(*t*) => +CLN1(*t*+1)		-SWI5(*t*) => +CLN1(*t*+1)	-SWI5(*t*) => +CLN1(*t*+1)
CLN2	G1	**+CLB1(*t*) => -CLN2(*t*+1)**	-SIC1(*t*-1) => -CLN2(*t*+1)	-SIC1(*t*-1) => -CLN2(*t*+1)	-SIC1(*t*-1) => -CLN2(*t*+1)	-SIC1(*t*-1) => -CLN2(*t*+1)
CLN3	M	+CDC53(*t*) => -CLN3(*t*+1)				
CLB1	M	**+CLB6(*t*) => -CLB1(*t*+1)**	+CLB6(*t*) => -CLB1(*t*+1)	**+CLB6(*t*) => -CLB1(*t*+1)**		-CLB5(*t*-4) => -CLB1(*t*+1)
CLB2	M	**+CLB6(*t*) => -CLB2(*t*+1)**	**+CLB6(*t*) => -CLB2(*t*+1)**	+CLB6(*t*) => -CLB2(*t*+1)		-CLB5(*t*-4) => -CLB2(*t*+1)
CLB4	G2	-CLN1(*t*) => -CLB4(*t*+1)	-CLN1(*t*) => -CLB4(*t*+1)			-CLB4(*t*-4) => +CLB4(*t*+1)
CLB5	G1					
CLB6	G1	-SWI5(*t*) => +CLB6(*t*+1)			+CLN3(*t*-3) => +CLB6(*t*+1)	-CDC20(*t*-4) => -CLB6(*t*+1)
MCM1						
SIC1	M/G1	+CLN3(*t*) => +SIC1(*t*+1)	+CLB2(*t*-1) => +SIC1(*t*+1)	-CLN2(*t*-2) => +SIC1(*t*+1)		**+CLB1(*t*-3) => +SIC1(*t*+1)**
SWI6		-SKP1(*t*)-CLN2(*t*) => -SWI6(*t*+1)				
CDC28		-MBP1(*t*) => +CDC28(*t*+1)				-CLB6(*t*-4) => +CDC28(*t*+1)
CDC53						
MBP1			+MCM1(*t*-1) => -MBP1(*t*+1)		-SKP1(*t*-3) => +MBP1(*t*+1)	
CDC34				-CDC34(t-2) => +CDC34(*t*+1)		-CDC34(*t*-4) => +CDC34(*t*+1)
SWI5	M	**+CLB1(*t*) => +SWI5(*t*+1)**	-SIC1(*t*-1) => +SWI5(*t*+1)	-SIC1(*t*-1) => +SWI5(*t*+1)	-SIC1(*t*-1) => +SWI5(*t*+1)	-SIC1(*t*-1) => +SWI5(*t*+1)
SKP1			-SWI6(*t*-1) => -SKP1(*t*+1)	+MBP1(*t*-2) => +SKP1(*t*+1)		
SWI4	M/G1	+CDC28(*t*) => +SWI4(*t*+1)		+CLB2(*t*-2) => +SWI4(*t*+1)	**+CLB2(*t*-2) => +SWI4(*t*+1)**	-CLB5(*t*-4) => +SWI4(*t*+1)
CDC20	M	-CLB1(*t*) => -CDC20(*t*+1)	**+CLB1(*t*-1) => +CDC20(*t*+1)**	+CLB1(*t*-1) => +CDC20(*t*+1)	-SWI6(*t*-3) => -CDC20(*t*+1)	-SWI6(*t*-3) => -CDC20(*t*+1)
HCT1						

**Table 2:** List of the identified time-delayed gene regulations for human Hela cell cycling. In the bold style are the rules extracted from confident trees and in the regular style are the rules extracted from putative trees.

Gene Name	Peak phase	Time Delayed				
		
		One-unit time	One-unit time	Three-unit time	Four-unit time	Five-unit time
PCNA	G1/S		+E2F1(*t*-1)+CCNA2(*t*-1)-CDC20(*t*-1) => +PCNA(*t*+1)			
NPAT	G1/S				**-CDC2(*t*-1) => +NPAT(*t*+1)**	
E2F1	G1/S	+BUB1B(*t*) => -E2F1(*t*+1)			+BUB1B(*t*) => -E2F1(*t*+1)	
CCNE1	G1/S	**+CDC2(*t*)-CDC25B(*t*) => +CCNE1(*t*+1)**	**+CDC2(*t*-1)+CDC20(*t*-1) => +CCNE1(*t*+1)**	**+CDC2(*t*-1)+CDC20(*t*-1) => +CCNE1(*t*+1)**	+CDC2(*t*-1)+CDC20(*t*-1) => +CCNE1(*t*+1)	-CCNF(*t*)-CDC20(*t*-4) => -CCNE1(*t*+1)
CDC25A	G1/S	-CCNE1(*t*)-CCNE1(*t*) +CDC20(*t*) => -CDC25A(*t*+1)			-CCNE1(*t*)-BUB1B(*t*-1) => +CDC25A(*t*+1)	-CCNE1(*t*)-STK15(*t*) => +CDC25A(*t*+1)
CDKN1A	G1/S	-CDKN1A(*t*)-DHFR(*t*) => +CDKN1A(*t*+1)	-CDKN1A(*t*)-DHFR(*t*-1) => +CDKN1A(*t*+1)	-CDKN1A(*t*)-BRCA1(*t*-2) => +CDKN1A(*t*+1)	-CDKN1A(*t*)+CDC20(*t*-3) => +CDKN1A(*t*+1)	-CDKN1A(*t*)+CDC20(*t*-3) => +CDKN1A(*t*+1)
BRCA1	S					
DHFR	S		**+BUB1B(*t*-1)+CDC25A(*t*-1) => +DHFR(*t*+1)**			
TYMS	S					
CCNF	G2	**+CDC2(*t*) -BUB1B(*t*) => -CCNF(*t*+1)**	+CDC2(*t*)-STK15(*t*-1) => -CCNF(*t*+1)	-CDC2(*t*)+CDC25C(*t*) => +CCNF(*t*+1)	**+CDC2(*t*) => +CCNF(*t*+1)**	**+CDC2(*t*) => +CCNF(*t*+1)**
CCNA2	G2	-CDC2(*t*)+STK15(*t*) => +CCNA2(*t*+1)	+CDC2(*t*)-STK15(*t*-1) => -CCNA2(*t*+1)		+CDC2(*t*) => +CCNA2(*t*+1)	**+CDC2(*t*) => +CCNA2(*t*+1)**
CDC20	G2/M	-STK15(*t*)-CKS2(*t*) => -CDC20(*t*+1)	**-STK15(*t*)+CDC20(*t*-1) => +CDC20(*t*+1)**	**-STK15(*t*)+CDC20(*t*-2) => +CDC20(*t*+1)**	-CKS2(*t*)-CDC20(*t*-3) => +CDC20(*t*+1)	+CCNE1(*t*)+TYMS(*t*) => +CDC20(*t*+1)
STK15	G2/M	**-CCNF(*t*)-CCNE1(*t*) => +STK15(*t*+1)**	**-CCNF(*t*)-CCNE1(*t*) => +STK15(*t*+1)**	**+CDC2(*t*-1)+CDC20(*t*-1) => -STK15(*t*+1)**	**-CDC2(*t*-1) => -STK15(*t*+1)**	**-CCNF(*t*) => -STK15(*t*+1)**
BUB1B	G2/M			+CDC2(*t*-1)+CDC20(*t*-1) => -BUB1B(*t*+1)	+CDC2(*t*-1)+CDC20(*t*-1) => -BUB1B(*t*+1)	+CDC2(*t*-1)+CDC20(*t*-1) => -BUB1B(*t*+1)
CKS2	G2/M	**-CCNF(*t*)-PCNA(*t*) => +CKS2(*t*+1)**	**+CCNA2(*t*-1)-BRCA1(*t*-1) => -CKS2(*t*+1)**	**+CCNA2(*t*-1)-BRCA1(*t*-1) => -CKS2(*t*+1)**	**+CCNA2(*t*-1)+CKS2(*t*-3) => -CKS2(*t*+1)**	-STK15(*t*) => -CKS2(*t*+1)
CDC25C	G2/M		+CDC2(*t*)+TYMS(*t*)-CDKN1A(*t*-1) => +CDC25C(*t*+1)	+CDC2(*t*)+TYMS(*t*)-CDKN1A(*t*-1) => +CDC25C(*t*+1)	**+CDC2(*t*)+TYMS(*t*)+PCNA(*t*-3) => +CDC25C(*t*+1)**	**+CDC2(*t*)+CDC25A(*t*-4)-CDC20(*t*-3) => +CDC25C(*t*+1)**
PLK	G2/M	**-STK15(*t*)-CCNF(*t*) => -PLK(*t*+1)**	**-STK15(*t*)-CCNF(*t*) => -PLK(*t*+1)**	**-STK15(*t*)-CCNF(*t*) => -PLK(*t*+1)**	**-STK15(*t*)-CCNF(*t*) => -PLK(*t*+1)**	**-STK15(*t*)-CKS2(*t*-4) => +PLK(*t*+1)**
CCNB1	G2/M					
CDC25B	G2/M	-CKS2(*t*)-CDKN1A(*t*)-BUB1B(*t*) => -CDC25B(*t*+1)	-CKS2(*t*)+PCNA(*t*-1) => +CDC25B(*t*+1)		**-CKS2(*t*)-CDC20(*t*-2) => +CDC25B(*t*+1)**	-CKS2(*t*)-CDC20(*t*-4) => +CDC25B(*t*+1)
CDC2	G2/M	-CDC2(*t*) => -CDC2(*t*+1)				-E2F1(*t*-4) => -CDC2(*t*+1)

**Table 3:** The *D*_*i *_= (*TdE C*_*i*_) matrix for the target gene *g*_*i*_. *g*_1_, …, *g*_*n *_are the putative regulating genes to be assessed. *d*_*kl *_values are the temporal transcriptions of these genes. *C*_*i*_denotes the phenotype (state) vector for the target gene *g*_*i *_at the temporal point (*T *+ 1, ..., *m*).

Gene\*t *+ 1	*g*_1_				…	*g*_*n*_				*C*_*i*_
				
	*t *- (*T *- 1)	…	*t *- 1	*t*		*t *- (*T *- 1)	…	*t *- 1	*t*	
*T *+ 1	*d*_11_	…	*d*_1(*T*-1)_	*d*_1*T*_	…	*d*_*n*1_	…	*d*_*n*(*T*-1)_	*d*_*nT*_	*C*_*i*(*T*+1)_
*T*+2	*d*_12_	…	*d*_1*T*_	*d*_1(*T*+1)_	…	*d*_*n*2_	…	*d*_*nT*_	*d*_*n*(*T*+1)_	*C*_*i*(*T*+2)_
…	…	…	…	…	…	…	…	…	…	…
*T *+ (*m *- *T*)	*d*_1(*m*-*T*)_	…	*d*_1(*m*-2)_	*d*_1(*m*-1)_	…	*d*_*n*(*m*-*T*)_	…	*d*_*n*(*m*-2)_	*d*_*n*(*m*-1)_	*C*_*im*_
