# Supplementary material for: Discovery of time-delayed gene regulatory networks based on temporal gene expression profiling
Source: BMC Bioinformatics. 2006 Jan 18;7:26. doi: 10.1186/1471-2105-7-26 (PMC1386718; doi:10.1186/1471-2105-7-26)
Supplement: Additional File 1 — The accuracy (%) of each gene's classifiers in all of the three estimations (cdc15, cdc28 and α-factor) at each delayed time point (T). [file 1471-2105-7-26-S1.pdf]

Additional file 1 (Table S1)– The accuracy (%) of each gene's classifiers in all of the three estimations (*cdc28*, *α-factor* and *cdc15*) at each delayed time point (*T*).

Gene Name	*T* = 1			*T* = 2			*T* = 3			*T* = 4			*T* = 5		
	cdc28	*α-factor*	*cdc15*	*cdc28*	*α-factor*	*cdc15*	*cdc28*	*α-factor*	*cdc15*	*cdc28*	*α-factor*	*cdc15*	*cdc28*	*α-factor*	*cdc15*
CLN1	75.00	70.60	60.00	73.30	56.20	45.00	57.10	66.70	45.00	76.90	92.90	60.00	75.00	92.30	40.00
CLN2	81.20	76.50	80.00	46.70	56.20	85.00	50.00	60.00	90.00	53.80	57.10	80.00	58.30	61.50	85.00
CLN3	50.00	23.50	80.00	46.70	25.00	55.00	42.90	53.30	50.00	53.80	21.40	50.00	66.70	61.50	60.00
CLB1	87.50	82.40	85.00	93.30	87.50	60.00	92.90	93.30	75.00	46.20	46.20	45.00	66.70	76.90	50.00
CLB2	87.50	88.20	80.00	93.30	93.70	85.00	92.90	100.00	65.00	46.20	64.30	45.00	75.00	69.20	60.00
CLB4	68.70	76.50	70.00	73.30	62.50	70.00	50.00	53.30	45.00	53.80	50.00	35.00	58.30	53.80	80.00
CLB5	50.00	58.80	65.00	53.30	62.50	50.00	42.90	66.70	60.00	53.80	57.10	45.00	25.00	69.20	40.00
CLB6	75.00	52.90	60.00	66.70	62.50	50.00	64.30	66.70	35.00	61.50	78.60	20.00	58.30	53.80	90.00
MCM1	31.20	47.10	30.00	60.00	25.00	30.00	57.10	53.30	45.00	53.80	50.00	45.00	58.30	69.20	15.00
SIC1	62.50	70.60	85.00	73.30	81.20	75.00	64.30	73.30	70.00	61.50	50.00	65.00	75.00	76.90	90.00
SWI6	50.00	47.10	75.00	46.70	43.70	40.00	64.30	40.00	55.00	61.50	35.70	45.00	25.00	46.20	60.00
CDC28	56.20	52.90	70.00	53.30	56.20	70.00	57.10	60.00	50.00	38.50	57.10	25.00	33.30	46.20	75.00
CDC53	62.50	35.30	50.00	40.00	62.50	60.00	35.70	60.00	55.00	69.20	42.90	55.00	41.70	46.20	30.00
MBP1	56.20	64.70	50.00	20.00	62.50	90.00	57.10	66.70	65.00	23.10	14.30	75.00	25.00	15.40	65.00
CDC34	37.50	35.30	60.00	33.30	37.50	65.00	35.70	53.30	75.00	69.20	42.90	65.00	41.70	61.50	85.00
SWI5	75.00	82.40	70.00	46.70	75.00	95.00	50.00	80.00	85.00	53.80	78.60	80.00	58.30	84.60	60.00
SKP1	25.00	41.20	65.00	33.30	31.20	75.00	42.90	53.30	75.00	38.50	57.10	65.00	41.70	46.20	50.00
SWI4	87.50	41.20	35.00	46.70	25.00	50.00	71.40	53.30	45.00	92.30	85.70	75.00	66.70	69.20	70.00
CDC20	62.50	76.50	70.00	73.30	75.00	75.00	64.30	73.30	70.00	76.90	57.10	65.00	75.00	53.80	55.00
HCT1	37.50	58.80	35.00	33.30	50.00	65.00	42.90	13.30	35.00	30.80	28.60	25.00	33.30	30.80	25.00

Note- The dark shaded cells meet the criterion for a certain tree. The light shaded cells are interpreted to be putative trees.
