# Supplementary material for: Discovery of time-delayed gene regulatory networks based on temporal gene expression profiling
Source: BMC Bioinformatics. 2006 Jan 18;7:26. doi: 10.1186/1471-2105-7-26 (PMC1386718; doi:10.1186/1471-2105-7-26)
Supplement: Additional File 2 — The biological support of the gene regulations for Yeast cell cycling. [file 1471-2105-7-26-S2.pdf]

Additional file 2 – The biological support of the gene regulations for Yeast cell cycling.

— Additional supporting analyses for the article: Xia Li, Shaoqi Rao, Wei Jiang, Chuanxing Li, Yun Xiao, Zheng Guo, Qingpu Zhang, Lihong Wang, Lei Du, Jing Li, Li Li, Tianwen Zhang and Qing K. Wang: **Discovery of time-delayed gene regulatory networks based on temporal gene expression profiling**. *BMC Bioinformatics* 2006, **7**.

Knowledge Databases

KEGG database

The KEGG [1, 2] is a suite of databases and associated software that integrates current knowledge on molecular interaction networks in biological processes (PATHWAY database), the information about the universe of genes and proteins (GENES/SSDB/KO databases), and the information about the universe of chemical compounds, drugs and their biochemical reactions (COMPOUND/DRUG/GLYCAN/REACTION databases). For our analyses we annotated results with PATHWAY database, which has 29,471 pathways generated from 245 reference pathways.

SGD and CYGD databases

SGD is a scientific database of the molecular biology and genetics of the yeast (*Saccharomyces cerevisiae*), which is commonly known as baker's or budding yeast. The MIPS Comprehensive Yeast Genome Database (CYGD) aims to present information on the molecular structure and functional network of the entirely sequenced, well-studied model eukaryote, the budding yeast (*Saccharomyces cerevisiae*). In addition, the data of various projects on related yeasts are used for comparative analysis. For our analyses we used these databases to annotate our results.

Results and Discussions

In total, we identified 32 statistically significant multiple-time-delayed gene regulations for *Saccharomyces Cerevisiae* cell cycling. Now, we subject the identified genetic relationships to biological verifications using the knowledge pools of KEGG, SGD, and CYGD databases. For this purpose, we defined three categories of biological evidence: supportive if there is explicit and direct experimental evidence demonstrating presence of such a regulatory relationship; predictive if previously documented evidence implies the possibilities of the regulatory interplays between the genes as defined in the multiple-time-delayed gene regulations, but the exact time-delayed mechanism(s) remains to be experimentally verified; new hypothetical if the biological knowledge for the regulation is totally lacking so far. After performing a comprehensive knowledge searching, we found that 72% of 32 uncovered relations (11 in supportive category and 12 in predictive category) are biologically sounding and documented previously, and the remaining 9 new hypothetical ones lacks knowledge so far (Table S2). However, we might miss some important biological evidence such as documented in other languages than Chinese and English, and unpublished results performed by individual labs. The knowledge for most of supportive relationships is mainly derived from and cross-verified by multiple sources (articles and databases). For example, for the mechanism that gene CLB1

inhibits gene CLN2, we found multiple lines of evidence in SGD database and PUBMED articles to support that the G2 cyclins CLB1P, CLB2P, CLB3P, and CLB4P inhibit CLN1 and CLN2 transcription.

Conclusions

Microarray technology has produced massive high-dimension data for life scientists to explore, analyze, and transform them into a mechanistic understanding. Our newly proposed algorithm TdGRN is specifically designed to reveal the time-delayed mechanisms of gene regulations using time series data. Our knowledge mining has proved that the gene regulations identified by TdGRN have strong evidence support, rendering the robust bioinformatics toolbox a promising and feasible computational approach to reversely engineering various biological networks.

References

1. Kanehisa M, Goto S: **KEGG: kyoto encyclopedia of genes and genomes**. *Nucleic Acids Res* 2000, **28**(1):27-30.
2. Kanehisa M: **A database for post-genome analysis**. *Trends Genet* 1997, **13**(9):375-376.
3. Amon A, Tyers M, Futcher B, Nasmyth K: **Mechanisms that help the yeast cell cycle clock tick: G2 cyclins transcriptionally activate G2 cyclins and repress G1 cyclins**. *Cell* 1993, **74**(6):993-1007.
4. Dirick L, Bohm T, Nasmyth K: **Roles and regulation of Cln-Cdc28 kinases at the start of the cell cycle of Saccharomyces cerevisiae**. *Embo J* 1995, **14**(19):4803-4813.
5. Stuart D, Wittenberg C: **CLN3, not positive feedback, determines the timing of CLN2 transcription in cycling cells**. *Genes Dev* 1995, **9**(22):2780-2794.
6. Tyers M, Tokiwa G, Futcher B: **Comparison of the Saccharomyces cerevisiae G1 cyclins: Cln3 may be an upstream activator of Cln1, Cln2 and other cyclins**. *Embo J* 1993, **12**(5):1955-1968.
7. Siegmund RF, Nasmyth KA: **The Saccharomyces cerevisiae Start-specific transcription factor Swi4 interacts through the ankyrin repeats with the mitotic Clb2/Cdc28 kinase and through its conserved carboxy terminus with Swi6**. *Mol Cell Biol* 1996, **16**(6):2647-2655.
8. Alexandru G, Zachariae W, Schleiffer A, Nasmyth K: **Sister chromatid separation and chromosome re-duplication are regulated by different mechanisms in response to spindle damage**. *Embo J* 1999, **18**(10):2707-2721.
9. Andrews B, Measday V: **The cyclin family of budding yeast: abundant use of a good idea**. *Trends Genet* 1998, **14**(2):66-72.
10. Willems AR, Lanker S, Patton EE, Craig KL, Nason TF, Mathias N, Kobayashi R, Wittenberg C, Tyers M: **Cdc53 targets phosphorylated G1 cyclins for degradation by the ubiquitin proteolytic pathway**. *Cell* 1996, **86**(3):453-463.
11. Nash P, Tang X, Orlicky S, Chen Q, Gertler FB, Mendenhall MD, Sicheri F, Pawson T, Tyers M: **Multisite phosphorylation of a CDK inhibitor sets a threshold for the onset of DNA replication**. *Nature* 2001, **414**(6863):514-521.
12. Kuhne C, Linder P: **A new pair of B-type cyclins from Saccharomyces cerevisiae that function early in the cell cycle**. *Embo J* 1993, **12**(9):3437-3447.
13. Dohrmann PR, Butler G, Tamai K, Dorland S, Greene JR, Thiele DJ, Stillman DJ: **Parallel pathways of gene regulation: homologous regulators SWI5 and ACE2**

differentially control transcription of HO and chitinase. *Genes Dev* 1992, **6**(1):93-104.
14. Grandin N, Reed SI: **Differential function and expression of Saccharomyces cerevisiae B-type cyclins in mitosis and meiosis**. *Mol Cell Biol* 1993, **13**(4):2113-2125.
15. Feldman RM, Correll CC, Kaplan KB, Deshaies RJ: **A complex of Cdc4p, Skp1p, and Cdc53p/cullin catalyzes ubiquitination of the phosphorylated CDK inhibitor Sic1p**. *Cell* 1997, **91**(2):221-230.

Table S2: Biological explanations of each regulation for yeast cell cycling genes.

Regulations	Category	Original	Description
+CLB1(t)=>-CLN2(t+1)	Supportive	[3-6]	The G2 cyclins CLB1P, CLB2P, CLB3P, and CLB4P inhibit CLN1 and CLN2 transcription.
+CDC28(t)=>+SWI4(t+1)	Supportive	[7]	SWI4 can be phosphorylated by CDC28P/CLB2P in vitro.
-SWI5(t)=>+CLB6(t+1)	Supportive	[KEGG sce:4110 (cell cycle)]	SWI5 regulates precise cell cycle timing of SIC1 expression. SIC1 can inhibit CDC28/CLB6 kinase.
-CLN1(t)=>-CLB4(t+1)	Supportive	[9]	The Cyclin/CDK complexes (CDC28/CLN1, CDC28/CLN2, and CDC28/CLN3) can regulate CLB proteolysis.
+CLN3(t-3)=>+CLB6(t+1)	Supportive	[9]	The Cyclin/CDK complexes (CDC28/CLN1, CDC28/CLN2, and CDC28/CLN3) can regulate CLB proteolysis.
-CLN2(t-2)=>+SIC1(t+1)	Supportive	[11]	Multisite phosphorylation by CDC28/CLN kinase targets SIC1P.
+CLN3(t)=>+SIC1(t+1)	Supportive	[CYGD]	SIC1 degradation depends on CLN3.
+CLB1(t)=>+SWI5(t+1)	Supportive	[KEGG SCE:4110 (CELL CYCLE)]	CDC28/CLB1 can regulate SWI5.
-CLB6(t-4)=>+CDC28(t+1)	Supportive	[12]	CLB6 can regulate CDC28P to promote initiation of DNA synthesis.
-CDC20(t-4)=>-CLB6(t+1)	Supportive	[8]	CDC20 serves as an activator of the APC and mediates ubiquitin-dependent protein degradation of PDS1P, and the cyclins CLB5P and CLB3P at the metaphase-to-anaphase transition of the cell cycle. CLB5 and CLB6 are closely related genes, so CDC20 maybe interact with CLB6.
-MBP1(t)=>+CDC28(t+1)	Supportive	[SGD]	CLB5 encodes a B-type cyclin that activates CDC28P to promote initiation of DNA synthesis. Both CLB5 and CLB6 promoters contain MCB (MluI cell cycle box) motifs, which are elements found in several DNA synthesis genes. The transcriptional activator MBF (MCB-binding factor), which is comprised of

			the MBP1 and SWI6 proteins, binding to the MCB elements to activate transcription.
+CLB1(t-3)=>+SIC1(t+1)	Predictive	[[KEGG SCE:4110 (CELL CYCLE)]]	SIC1 inhibits CLB1.
+SWI5(t)=>-CLN1(t+1)	Predictive	[13], [[KEGG SCE:4110 (CELL CYCLE)]]	SWI5 encodes a transcription factor that activates transcription of genes expressed in G1 phase of the cell cycle. SWI5 regulates precise cell cycle timing of SIC1 expression. CLN1 encodes a G1 cyclin involved in regulation of the cell cycle. CDC28/CLN1 phosphorylate SIC1.
+CLB6(t)=>-CLB1(t+1)	Predictive	[12, 14]	CLB1, CLB2, CLB5, and CLB6 are all B-type Cyclins. CLB1 and CLB2 activate CDC28P to promote the transition from G2 to M phase. CLB5 and CLB6 activate CDC28P to promote initiation of DNA synthesis.
+CLB6(t)=>-CLB2(t+1)	Predictive	[12, 14]	CLB1, CLB2, CLB5, and CLB6 are all B-type Cyclins. CLB1 and CLB2 activate CDC28P to promote the transition from G2 to M phase. CLB5 and CLB6 activate CDC28P to promote initiation of DNA synthesis.
-CLB5(t-4)=>-CLB1(t+1)	Predictive	[12, 14]	CLB1, CLB2, CLB5, and CLB6 are all B-type Cyclins. CLB1 and CLB2 activate CDC28P to promote the transition from G2 to M phase. CLB5 and CLB6 activate CDC28P to promote initiation of DNA synthesis.
-CLB5(t-4)=>-CLB2(t+1)	Predictive	[12, 14]	CLB1, CLB2, CLB5, and CLB6 are all B-type Cyclins. CLB1 and CLB2 activate CDC28P to promote the transition from G2 to M phase. CLB5 and CLB6 activate CDC28P to promote initiation of DNA synthesis.
-SWI6(t-3)=>-CDC20(t+1)	Predictive	[[KEGG SCE:4110 (CELL CYCLE)]]	Both SWI6 and CDC20 can interact with CLB5/CDC28 and CLB6/CDC28 kinase complexes.
+CLB2(t-1)=>+SIC1(t+1)	Predictive	[15]	SIC1 is the inhibitor of CDC28/CLB kinase complex that controls the G1/S phase transition, preventing premature S phase entry and ensuring genomic integrity
+CDC53(t)=>-CLN3(t+1)	Predictive	[10]	CDC53 is a subunit of structural protein of SCF complex, which also contains SKP1P, CDC34P, and an F-box protein, involved in ubiquitination; SCF promotes the G1-S

			transition by targeting G1 cyclins.
-SIC1(t-1)=>+SWI5(t+1)	Predictive	[CYGD]	SWI5P is required for precise cell cycle timing of SIC1 expression.
-CLB5(t－4)=>+SWI4(t+1)	Predictive	[[KEGG SCE:4110 (CELL CYCLE)]]	SWI4 is DNA binding component of the SBF complex (SWI4P/SWI6P), and can bind to SCB promoter. CLN1 and CLN2 bind with CDC28, form kinase complex, which can phosphorylate SIC1. SIC1 can inhibit CLB5.
-SIC1(t-1)=>-CLN2(t+1)	Predictive	[11]	Multisite phosphorylation by CDC28/CLN kinase targets SIC1P.
-SKP1(t)-CLN2(t)=> -SWI6(t+1)	New hypothetical		
+MCM1(t-1)=>-MBP1(t+1)	New hypothetical		
-SKP1(t-3)=>+MBP1(t+1)	New hypothetical		
-CDC34(t-2) =>+CDC34(t+1)	New hypothetical		
+CLB2(t-2)=>+SWI4(t+1)	New hypothetical		
-CLB1(t)=>-CDC20(t+1)	New hypothetical		
-CLB4(t－4)=>+CLB4(t+1)	New hypothetical		
-SWI6(t-1)=>-SKP1(t+1)	New hypothetical		
+MBP1(t-2)=>+SKP1(t+1)	New hypothetical		
