# Supplementary material for: Discovery of time-delayed gene regulatory networks based on temporal gene expression profiling
Source: BMC Bioinformatics. 2006 Jan 18;7:26. doi: 10.1186/1471-2105-7-26 (PMC1386718; doi:10.1186/1471-2105-7-26)
Supplement: Additional File 3 — The accuracy (%) of each gene's classifiers in all of the three estimations (Thy-Thy2, Thy-Noc and Thy-Thy3) at each delayed time point (T). [file 1471-2105-7-26-S3.pdf]

Additional file 3 (Table S3) – The accuracy (%) of each gene's classifiers in all of the three estimations (*Thy-Thy2*, *Thy-Noc* and *Thy-Thy3*) at each delayed time point (*T*).

Gene name	*T* = 1			*T* = 2			*T* = 3			*T* = 4			*T* = 5		
	Thy-Thy2	*Thy-Noc*	*Thy-Thy3*	*Thy-Thy2*	*Thy-Noc*	*Thy-Thy3*	*Thy-Thy2*	*Thy-Noc*	*Thy-Thy3*	*Thy-Thy2*	*Thy-Noc*	*Thy-Thy3*	*Thy-Thy2*	*Thy-Noc*	*Thy-Thy3*
PCNA	66.67	44.44	65.99	72.73	35.29	68.74	40.00	50.00	68.22	66.67	53.33	68.64	50.00	42.86	70.19
NPAT	50.00	55.56	71.61	45.45	52.94	69.78	70.00	62.50	72.19	77.78	66.67	76.94	62.50	57.14	77.59
E2F1	75.00	66.67	78.75	54.55	52.94	75.33	60.00	50.00	77.03	66.67	66.67	74.71	62.50	57.14	79.98
CCNE1	91.67	72.22	81.11	90.91	76.47	80.52	90.00	81.25	79.56	88.89	73.33	76.38	87.50	57.14	79.56
CDC25A	91.67	50.00	67.81	63.64	47.06	67.63	70.00	56.25	65.60	77.78	53.33	68.94	75.00	71.43	65.75
CDKN1A	41.67	77.78	77.26	72.73	88.24	81.78	50.00	81.25	82.73	55.56	80.00	79.18	50.00	85.71	77.02
BRCA1	33.33	44.44	62.96	63.64	58.82	58.67	50.00	50.00	56.97	33.33	60.00	57.20	37.50	50.00	62.82
DHFR	50.00	50.00	58.13	72.73	76.47	62.52	50.00	56.25	59.69	55.56	60.00	55.28	75.00	64.29	53.94
TYMS	41.67	77.78	54.69	36.36	88.24	56.96	50.00	68.75	56.30	55.56	46.67	58.27	50.00	50.00	60.36
CCNF	66.67	72.22	73.36	81.82	58.82	76.30	90.00	62.50	77.96	88.89	80.00	79.39	87.50	78.57	76.86
CCNA2	75.00	88.89	81.68	72.73	64.71	83.33	70.00	62.50	81.52	66.67	86.67	83.63	87.50	85.71	85.93
CDC20	83.33	61.11	68.92	81.82	82.35	68.44	90.00	81.25	65.34	88.89	53.33	67.50	100.00	42.86	72.18
STK15	100.00	77.78	83.84	100.00	82.35	83.56	90.00	81.25	83.78	100.00	80.00	86.06	100.00	85.71	85.63
BUB1B	41.67	50.00	71.61	36.36	47.06	73.26	80.00	68.75	80.94	77.78	73.33	75.19	62.50	71.43	74.21
CKS2	83.33	77.78	79.40	90.91	76.47	78.37	90.00	81.25	78.51	88.89	80.00	75.28	62.50	71.43	78.18
CDC25C	50.00	61.11	73.01	63.64	76.47	72.67	70.00	81.25	70.62	77.78	66.67	70.91	75.00	71.43	72.92
PLK	91.67	83.33	93.93	90.91	82.35	88.89	90.00	81.25	88.07	100.00	86.67	84.33	100.00	92.86	89.42
CCNB1	58.33	61.11	59.99	63.64	70.59	57.11	80.00	62.50	58.45	66.67	80.00	59.07	62.50	71.43	57.88
CDC25B	83.33	66.67	67.69	90.91	47.06	73.70	80.00	62.50	70.15	77.78	80.00	67.73	50.00	85.71	74.09
CDC2	58.33	72.22	78.08	63.64	52.94	81.70	60.00	50.00	78.32	55.56	53.33	75.61	100.00	42.86	84.30

Note- The dark shaded cells meet the criterion for a certain tree. The light shaded cells are interpreted to be putative trees.
