# Supplementary material for: Discovery of time-delayed gene regulatory networks based on temporal gene expression profiling
Source: BMC Bioinformatics. 2006 Jan 18;7:26. doi: 10.1186/1471-2105-7-26 (PMC1386718; doi:10.1186/1471-2105-7-26)
Supplement: Additional File 4 — The biological support of the gene regulations for human HeLa cell cycling. [file 1471-2105-7-26-S4.pdf]

Additional file 4 – The biological support of the gene regulations for human HeLa cell cycling.

— Additional supporting analyses for the article: Xia Li, Shaoqi Rao, Wei Jiang, Chuanxing Li, Yun Xiao, Zheng Guo, Qingpu Zhang, Lihong Wang, Lei Du, Jing Li, Li Li, Tianwen Zhang and Qing K. Wang: **Discovery of time-delayed gene regulatory networks based on temporal gene expression profiling**. *BMC Bioinformatics* 2006, **7**.

Materials

In the additional report for the further detailed biological explanations of each regulation for 20 Hela cell cycling genes, we present and discuss the results from analysis of four web resources: Entrez Gene, PUBMED, KEGG and BIND.

Description of Entrez Gene

Entrez Gene is one of the several gene-centered resources at NCBI [1], which is publicly available at http://www.ncbi.nlm.nih.gov/entrez/query.fcgi?db=gene. Entrez Gene has been implemented at the National Center for Biotechnology Information (NCBI) to organize information about genes, serving as a major node in the nexus of genomic map, sequence, expression, protein structure, function, and homology data. Each Entrez Gene record is assigned a unique identifier, the GeneID that can be tracked through revision cycles. Entrez Gene records are established for known or predicted genes, which are defined by nucleotide sequence or map position, and have links to Entrez Nucleotide, Entrez Protein, PubMed, OMIM, BIND, KEGG, CDD, dbSNP, GENSAT, GEO, HomoloGene, UniGene, and UniSTS etc. Entrez Gene is an Integrated Access to Genes of Genomes in the Reference Sequence Collection.

Description of PUBMED

PubMed, publicly available at http://www.ncbi.nlm.nih.gov/entrez/query.fcgi?db=PubMed, is a free resource. It was developed and maintained by the National Center for Biotechnology Information (NCBI) at the National Library of Medicine (NLM) located at the National Institutes of Health. PubMed lets a user search more than 15 million bibliographic citations and abstracts in the fields of medicine, nursing, dentistry, veterinary medicine, the health care system, and preclinical sciences. It provides access to MEDLINE® and to articles in selected life sciences journals not included in MEDLINE.

Description of KEGG

The KEGG database [2, 3] is a suite of databases and associated software to integrate current knowledge on molecular interaction networks in biological processes (PATHWAY database), the information about the universe of genes and proteins (GENES/SSDB/KO databases), and the information about the universe of chemical compounds, drugs and their biochemical reactions (COMPOUND/DRUG/GLYCAN/REACTION databases).

Description of BIND

The Biomolecular Interaction Network Database (BIND), publicly available at http://bind.ca/Action, is a collection of records documenting molecular interactions [4]. The contents of BIND include high-throughput data submissions and hand-curated information gathered from the scientific literature. BIND is an interaction database with three classifications for molecular associations: molecules that associate with each other to form interactions, molecular complexes that are formed from one or more interaction(s) and pathways that are defined by a specific sequence of two or more interactions. A BIND record represents an interaction between two or more objects that is believed to occur in a living organism. A biological object can be a protein, DNA, RNA, ligand, molecular complex, gene, photon or an unclassified biological entity. BIND records are created for interactions, which have been shown experimentally and published in at least one peer-reviewed journal. A record also references any papers with experimental evidence that support or dispute the associated interaction.

Results and Discussions

In our work, we uncovered 58 regulation relationships through constructing the time-delayed gene regulatory networks for 20 Hela cell cycling genes. Considering 24 overlapping ones, there are 44 unique relationships. The detailed explanation for each time-delayed regulation is given in Table S4. We annotated the regulations with PUBMED, Entrez Gene, BIND and KEGG databases. For this purpose, we defined three categories of biological evidence: supportive if there is explicit and direct experimental evidence demonstrating presence of such a regulatory relationship; predictive if previous documented evidence implies the possibilities of the regulatory interplays between the genes as defined in the multiple-time-delayed gene regulation, but the exact time-delayed mechanism(s) remains to be experimentally verified; new hypothetical if the biological knowledge for the regulation is totally lacking so far. Based on this classification, 18 time-delayed regulations are supportive, 10 predictive and 16 novel hypothetical. Therefore, 64% of the uncovered relations (18 supportive and 10 predictive) are biologically sounding and documented previously.

The most striking consistency is our discoveries of the highly confident regulations for E2F1, the cdk/cyclin complexes genes (CDC2, CCNA2 and CCNB1) and STK15. The protein encoded by the gene E2F1 is a member of the E2F family of transcription factors. The E2F family plays a crucial role in the control of cell cycle and action of tumor suppressor proteins and is also a target of the transforming proteins of small DNA tumor viruses. This protein and another 2 members, E2F2 and E2F3, have an additional cyclin binding domain. It is the transcription factor of six of the 20 Hela cell cycling genes, including itself. Here, we successfully verified the direct regulatory relationships of E2F1-CDC2 [5], E2F1-CCNA2 [5, 6], E2F1-BRCA1[6, 7], and several cooperative relationships among them.

The cdk/cyclin complexes play an important role in cell cycle regulation. Here, three genes CDC2, CCNA2 and CCNB1 are investigated. The protein encoded by the gene CDC2 is a member of the Ser/Thr protein kinase family. This protein is a catalytic subunit of the highly conserved protein kinase complex known as M-phase promoting factor (MPF), which is essential for G1/S and G2/M phase transitions of eukaryotic cell cycle. Mitotic cyclins stably associate with this protein and function as regulatory subunits. The kinase activity of this protein is controlled by cyclin accumulation and destruction through the cell cycle. The phosphorylation and dephosphorylation of this protein also play important regulatory roles in cell cycle control. The protein encoded by the gene CCNA2 belongs to the highly conserved cyclin family, whose

members are characterized by a dramatic periodicity in protein abundance through the cell cycle. Cyclins function as regulators of CDK kinases. Different cyclins exhibit distinct expression and degradation patterns which contribute to the temporal coordination of each mitotic event. In contrast to cyclin A1, which is present only in germ cells, CCNA2 is expressed in various tissues. CCNA2 binds and activates CDC2 or CDK2 kinases, and thus promotes both cell cycle G1/S and G2/M transitions. The protein encoded by the gene CCNB1 is a regulatory protein involved in mitosis. The gene product complexes with p34(CDC2) to form the maturation-promoting factor (MPF). Two alternative transcripts have been found, a constitutively expressed transcript and a cell cycle-regulated transcript, which is expressed predominantly during G2/M phase. The different transcripts result from the use of alternate transcription initiation sites. It is also known that: CDC2 can interact with E2F1 [5], CCNA2 [8], CCNB1, CDC25A, CDC25B, CDC25C and CDKN1A; CCNA2 can interact with E2F1 [5, 6], CDC2 [8], and CDKN1A; and CCNB1 can interact with CDC2, CCNF, BRCA1 and CDKN1A. Not surprisingly, most of these relationships are captured in our gene networking analyses.

The protein encoded by the gene STK15 is a cell cycle-regulated kinase that appears to be involved in microtubule formation and/or stabilization at the spindle pole during chromosome segregation. It is related to CDC2 and CDC20. Crane and copartners [9] found “Cdh1 (CDC2)-activated form of the APC/C is responsible for destruction of Aur-A (STK15) during the somatic cell cycle in vivo”. Du and Hannon [10] revealed that “The activity of STK15 is regulated by phosphorylation and ubiquitin-mediated degradation, and physically interacts with protein phosphatase 1 (PP1) and CDC20” in their work.

Conclusions

Again, as in the biological support analysis for Yeast cell cycling, we have demonstrated that most of the identified time-delayed gene regulations (up to 64%) have convincing experimental evidence. The remaining regulations can be treated as the novel hypotheses and may define novel genetic pathways. We conclude that the successful applications of the proposed TdGRN algorithms to two cell cycling systems have proved its power and robustness to generate a working blueprint for mapping dynamic mechanisms of gene regulations in the four-dimension space of biological entity and time.

References

1. Maglott D, Ostell J, Pruitt KD, Tatusova T: **Entrez Gene: gene-centered information at NCBI**. *Nucleic Acids Res* 2005, **33**(Database issue):D54-58.
2. Kanehisa M: **A database for post-genome analysis**. *Trends Genet* 1997, **13**(9):375-376.
3. Kanehisa M, Goto S: **KEGG: kyoto encyclopedia of genes and genomes**. *Nucleic Acids Res* 2000, **28**(1):27-30.
4. Alfarano C, Andrade CE, Anthony K, Bahroos N, Bajec M, Bantoft K, Betel D, Bobechko B, Boutilier K, Burgess E *et al*: **The Biomolecular Interaction Network Database and related tools 2005 update**. *Nucleic Acids Res* 2005, **33**(Database issue):D418-424.
5. Takahashi Y, Rayman JB, Dynlacht BD: **Analysis of promoter binding by the E2F and pRB families in vivo: distinct E2F proteins mediate activation and repression**. *Genes Dev* 2000, **14**(7):804-816.

6. Hernando E, Nahle Z, Juan G, Diaz-Rodriguez E, Alaminos M, Hemann M, Michel L, Mittal V, Gerald W, Benezra R *et al*: **Rb inactivation promotes genomic instability by uncoupling cell cycle progression from mitotic control**. *Nature* 2004, **430**(7001):797-802.
7. Hayami R, Sato K, Wu W, Nishikawa T, Hiroi J, Ohtani-Kaneko R, Fukuda M, Ohta T: **Down-regulation of BRCA1-BARD1 ubiquitin ligase by CDK2**. *Cancer Res* 2005, **65**(1):6-10.
8. Lees EM, Harlow E: **Sequences within the conserved cyclin box of human cyclin A are sufficient for binding to and activation of cdc2 kinase**. *Mol Cell Biol* 1993, **13**(2):1194-1201.
9. Crane R, Kloepfer A, Ruderman JV: **Requirements for the destruction of human Aurora-A**. *J Cell Sci* 2004, **117**(Pt 25):5975-5983.
10. Du J, Hannon GJ: **The centrosomal kinase Aurora-A/STK15 interacts with a putative tumor suppressor NM23-H1**. *Nucleic Acids Res* 2002, **30**(24):5465-5475.
11. Russo AA, Jeffrey PD, Pavletich NP: **Structural basis of cyclin-dependent kinase activation by phosphorylation**. *Nat Struct Biol* 1996, **3**(8):696-700.
12. Demetrick DJ, Zhang H, Beach DH: **Chromosomal mapping of the human genes CKS1 to 8q21 and CKS2 to 9q22**. *Cytogenet Cell Genet* 1996, **73**(3):250-254.
13. Liu J, Schmitz JC, Lin X, Tai N, Yan W, Farrell M, Bailly M, Chen T, Chu E: **Thymidylate synthase as a translational regulator of cellular gene expression**. *Biochim Biophys Acta* 2002, **1587**(2-3):174-182.
14. St Clair S, Giono L, Varmeh-Ziaie S, Resnick-Silverman L, Liu WJ, Padi A, Dastidar J, DaCosta A, Mattia M, Manfredi JJ: **DNA damage-induced downregulation of Cdc25C is mediated by p53 via two independent mechanisms: one involves direct binding to the cdc25C promoter**. *Mol Cell* 2004, **16**(5):725-736.
15. Tang Z, Shu H, Oncel D, Chen S, Yu H: **Phosphorylation of Cdc20 by Bub1 provides a catalytic mechanism for APC/C inhibition by the spindle checkpoint**. *Mol Cell* 2004, **16**(3):387-397.
16. Lampson MA, Kapoor TM: **The human mitotic checkpoint protein BubR1 regulates chromosome-spindle attachments**. *Nat Cell Biol* 2005, **7**(1):93-98.
17. Poon RY, Jiang W, Toyoshima H, Hunter T: **Cyclin-dependent kinases are inactivated by a combination of p21 and Thr-14/Tyr-15 phosphorylation after UV-induced DNA damage**. *J Biol Chem* 1996, **271**(22):13283-13291.
18. Sekiguchi T, Hunter T: **Induction of growth arrest and cell death by overexpression of the cyclin-Cdk inhibitor p21 in hamster BHK21 cells**. *Oncogene* 1998, **16**(3):369-380.
19. Mattock H, Lane DP, Warbrick E: **Inhibition of cell proliferation by the PCNA-binding region of p21 expressed as a GFP miniprotein**. *Exp Cell Res* 2001, **265**(2):234-241.
20. Pines J, Hunter T: **The differential localization of human cyclins A and B is due to a cytoplasmic retention signal in cyclin B**. *Embo J* 1994, **13**(16):3772-3781.
21. Kong M, Barnes EA, Ollendorff V, Donoghue DJ: **Cyclin F regulates the nuclear localization of cyclin B1 through a cyclin-cyclin interaction**. *Embo J* 2000, **19**(6):1378-1388.

22. Sebastian B, Kakizuka A, Hunter T: **Cdc25M2 activation of cyclin-dependent kinases by dephosphorylation of threonine-14 and tyrosine-15**. *Proc Natl Acad Sci U S A* 1993, **90**(8):3521-3524.
23. Sengupta S, Shimamoto A, Koshiji M, Pedeux R, Rusin M, Spillare EA, Shen JC, Huang LE, Lindor NM, Furuichi Y *et al*: **Tumor suppressor p53 represses transcription of RECQ4 helicase**. *Oncogene* 2005, **24**(10):1738-1748.
24. Park KK, Rue SW, Lee IS, Kim HC, Lee IK, Ahn JD, Kim HS, Yu TS, Kwak JY, Heintz NH *et al*: **Modulation of Sp1-dependent transcription by a cis-acting E2F element in dhfr promoter**. *Biochem Biophys Res Commun* 2003, **306**(1):239-243.
25. Kraft C, Vodermaier HC, Maurer-Stroh S, Eisenhaber F, Peters JM: **The WD40 propeller domain of Cdh1 functions as a destruction box receptor for APC/C substrates**. *Mol Cell* 2005, **18**(5):543-553.
26. Bates GJ, Nicol SM, Wilson BJ, Jacobs AM, Bourdon JC, Wardrop J, Gregory DJ, Lane DP, Perkins ND, Fuller-Pace FV: **The DEAD box protein p68: a novel transcriptional coactivator of the p53 tumour suppressor**. *Embo J* 2005, **24**(3):543-553.
27. Wei X, Xu H, Kufe D: **Human MUC1 oncoprotein regulates p53-responsive gene transcription in the genotoxic stress response**. *Cancer Cell* 2005, **7**(2):167-178.
28. Zhang H, Somasundaram K, Peng Y, Tian H, Bi D, Weber BL, El-Deiry WS: **BRCA1 physically associates with p53 and stimulates its transcriptional activity**. *Oncogene* 1998, **16**(13):1713-1721.

Table S4: Biological explanations of each regulation for 20 Hela cell cycling genes.

Regulations	Category	Original	Description
-E2F1(t-4)=>-CDC2(t+1)	Supportive	[5]	E2F1 interacts with the CDC2 promoter. E2F1 is a transcription factor of CDC2, and regulates the expression of CDC2 [5].
+CDC2(t-1)+CDC20(t-1) =>-STK15(t+1)	Supportive	[9, 10]	Crane and copartners found that “Cdh1 (CDC2)-activated form of the APC/C is responsible for destruction of Aur-A (STK15) during the somatic cell cycle in vivo.” [9].<br>Du and Hannon exposed that “The activity of STK15 is regulated by phosphorylation and ubiquitin-mediated degradation, and physically interacts with protein phosphatase 1 (PP1) and CDC20” in their work [10].
+CDC2(t)-STK15(t-1) =>-CCNA2(t+1)	Supportive	[8, 9]	Crane and copartners found that “Cdh1 (CDC2)-activated form of the APC/C is responsible for destruction of Aur-A (STK15) during the somatic cell cycle in vivo.” [9].<br>The cyclin-dependent kinase CDC2 (cdc2) interacts with cyclin A2 (CCNA2) [8].
-STK15(t)-CKS2(t) =>-CDC20(t+1)	Supportive	[10]	Du and Hannon exposed that “The activity of STK15 is regulated by phosphorylation and ubiquitin-mediated degradation, and physically interacts with protein phosphatase 1 (PP1) and CDC20” in their work [10].
+CCNA2(t-1)+CKS2(t-3) =>-CKS2(t+1)	Supportive	[11, 12]	Cyclin A2 (CCNA2) interacts with CDK2 [11]. “The human cdk2/cyclin A kinase complex is a key regulator of the events of S phase. This complex contains several proteins involved in regulating its

			catalytic activity, including one or more of the CKS proteins, which have recently been shown to inhibit the activation of the cdk2 kinase." [12].<br>This regulation also implies CKS2 itself may regulate transcription.
-CDC2(t-1)=>-STK15(t+1)	Supportive	[9]	Crane and copartners found that "Cdh1 (CDC2)-activated form of the APC/C is responsible for destruction of Aur-A (STK15) during the somatic cell cycle in vivo." [9].
+CDC2(t)=>+CCNA2(t+1)<br>(2 same regulations in results)	Supportive	[8]	The cyclin-dependent kinase CDC2 (cdc2) interacts with cyclin A2 (CCNA2) [8].
-CDC2(t)+STK15(t)<br>=>+CCNA2(t+1)	Supportive	[8, 9]	Crane and copartners found that "Cdh1 (CDC2)-activated form of the APC/C is responsible for destruction of Aur-A (STK15) during the somatic cell cycle in vivo." [9].<br>The cyclin-dependent kinase CDC2 (cdc2) interacts with cyclin A2 (CCNA2) [8].
-STK15(t)+CDC20(t-1)<br>=>+CDC20(t+1)	Supportive	[10]	Du and Hannon exposed that "The activity of STK15 is regulated by phosphorylation and ubiquitin-mediated degradation, and physically interacts with protein phosphatase 1 (PP1) and CDC20" in their work [10].<br>This regulation also implies CDC20 itself may regulate transcription.
-STK15(t) +CDC20(t-2)<br>=>+CDC20(t+1)	Supportive	[10]	Du and Hannon exposed that "The activity of STK15 is regulated by phosphorylation and ubiquitin-mediated degradation, and physically interacts with protein phosphatase 1 (PP1) and CDC20" in their work [10].
+CDC2(t)+TYMS(t)<br>-CDKN1A(t-1)<br>=>+CDC25C(t+1)<br>(2 same regulations in results)	Supportive	[13, 14], [KEGG hsa:4110 (cell cycle)]	In the map hsa:4110 (cell cycle) from KEGG, CDC25C can dephosphorylate CDC2.<br>Liu and collaborators found that "There is evidence that TS (TYMS), in addition to interacting with its own TS mRNA, forms a ribonucleoprotein complex with a number of other cellular mRNAs, including those corresponding to the p53 tumor suppressor gene and the myc family of transcription factors." [13]. P53 interacts with the CDC25C promoter [14]. It is suggested that TYMS may regulate CDC25C through P53.
+CDC2(t)+TYMS(t)<br>+PCNA(t-3)=>+CDC25C(t+1)	Supportive	[13, 14], [KEGG hsa:4110 (cell cycle)]	In the map hsa:4110 (cell cycle) from KEGG, CDC25C can dephosphorylate CDC2.<br>Liu and collaborators found that "There is evidence that TS (TYMS), in addition to interacting with its own TS mRNA, forms a ribonucleoprotein complex with a number of other cellular mRNAs, including those corresponding to the p53 tumor suppressor gene and the myc family of transcription factors." [13]. P53 interacts with the CDC25C promoter [14]. It is suggested that TYMS may regulate CDC25C through P53.

+CDC2(t)+CDC25A(t-4) -CDC20(t-3) =>+CDC25C(t+1)	Supportive	[KEGG hsa:4110 (cell cycle)]	In the map hsa:4110 (cell cycle) from KEGG, both CDC25A and CDC25C can dephosphorylate CDC2.<br>It is suggested that CDC25A may regulate CDC25C through CDC2.
+CDC2(t-1)+CDC20(t-1) =>-BUB1B(t+1)<br>(3 same regulations in results)	Supportive	[9, 15, 16]	Bub1 directly phosphorylates Cdc20 in vitro and inhibits the ubiquitin ligase activity of APC/C(Cdc20) catalytically [15].<br>Crane and copartners found that "Cdh1 (CDC2)-activated form of the APC/C is responsible for destruction of Aur-A (STK15) during the somatic cell cycle in vivo." [9]. STK15 interacts with BUB1B [16]. It is suggested that CDC2 may relate to BUB1B through STK15.
+E2F1(t-1)+CCNA2(t-1) -CDC20(t-1) =>+PCNA(t+1)	Supportive	[5, 6, 17-19]	E2F1 interacts with the CCNA2 promoter. E2F1 is a transcription factor of CCNA2, and regulates the expression of CCNA2 [5, 6].<br>Both CCNA2 and PCNA can interact with CDKN1A (p21) [17-19]. The interaction between PCNA and CDKN1A was modeled on a demonstrated interaction between human p21 and hamster PCNA [18]. It is suggested that CCNA2 may relate to PCNA through CDKN1A.
+CDC2(t)-STK15(t-1) =>-CCNF(t+1)	Supportive	[9, 20-22]	Crane and copartners found that "Cdh1 (CDC2)-activated form of the APC/C is responsible for destruction of Aur-A (STK15) during the somatic cell cycle in vivo." [9].<br>Both CDC2 (CDK1) and CCNF interact with CCNB1 (cyclin B1) [20-22]. CDC2 interacts with CCNB1 (cyclin B1) to form a complex [22] and this interaction was modeled on a demonstrated interaction between monkey CDC2 and human CCNB1 [20]. It is suggested that CDC2 may relate to CCNF through CCNB1.
+CDC2(t)-CDC25B(t) =>+CCNE1(t+1)	Supportive	[KEGG hsa:4110 (cell cycle)]	In the map hsa:4110 (cell cycle) from KEGG, CDC25B can dephosphorylate CDC2.
+CCNA2(t-1)-BRCA1(t-1) =>-CKS2(t+1)<br>(2 same regulations in results)	Supportive	[6-8]	Wang and copartners suggested that "BRCA1 could be an important negative regulator of cell cycle that functions through interaction with E2F transcriptional factors and phosphorylation by cyclins/cdk complexes with the zinc ring finger functioning as a major protein-protein interaction domain."[7]. The cyclin-dependent kinase CDC2 (cdc2) interacts with cyclin A2 (CCNA2) to form a complex [8]. And CCNA2 and BRCA1 share a common transcription factor E2F1[6, 7].
-CDKN1A(t)-DHFR(t-1) =>+CDKN1A(t+1)<br>(2 same regulations in results)	Predictive	[23, 24]	CDKN1A and DHFR share a common transcription factor SP1[23, 24] It is suggested that CDKN1A may relate to DHFR through SP1.<br>This regulation also implies CKS2 itself may regulate transcription.

-CDC2(t-1)=>+NPAT(t+1)	Predictive	[5, 24]	E2F1 interacts with the CDC2 and NAPT promoter.E2F1 is a transcription factor of CDC2 and NAPT, and regulates the expression of CDC2 and NAPT [5, 24]. So CDC2 and NAPT share a common transcription factor E2F1.
+BUB1B(t-1)+CDC25A(t-1) =>+DHFR(t+1)	Predictive	[5, 24]	E2F1 interacts with the CDC25A and DHFR promoter.E2F1 is a transcription factor of CDC25A and DHFR, and regulates the expression of CDC25A and DHFR [5, 24]. So CDC25A and DHFR share a common transcription factor E2F1.
-CCNF(t)-CCNE1(t) =>+STK15(t+1) (2 same regulations in results)	Predictive	[9, 20-22]	Both CDC2 (CDK1) and CCNF interact with CCNB1 (cyclin B1) [20-22]. CDC2 interacts with CCNB1 (cyclin B1) to form a complex [22] and this interaction was modeled on a demonstrated interaction between monkey CDC2 and human CCNB1 [20].<br>Crane and copartners found that “Cdh1 (CDC2)-activated form of the APC/C is responsible for destruction of Aur-A (STK15) during the somatic cell cycle in vivo.” [9].<br>It is suggested that STK15 may relate to CCNF through CCNB1 and CDC2.
-CCNF(t)=>-STK15(t+1)	Predictive	[9, 20-22]	Both CDC2 (CDK1) and CCNF interact with CCNB1 (cyclin B1) [20-22]. CDC2 interacts with CCNB1 (cyclin B1) to form a complex [22] and this interaction was modeled on a demonstrated interaction between monkey CDC2 and human CCNB1 [20].<br>Crane and copartners found that “Cdh1 (CDC2)-activated form of the APC/C is responsible for destruction of Aur-A (STK15) during the somatic cell cycle in vivo.” [9].<br>It is suggested that STK15 may relate to CCNF through CCNB1 and CDC2.
+CDC2(t-1)+CDC20(t-1) =>+CCNE1(t+1) (3 same regulations in results)	Predictive	[20, 22, 25]	Both CDC2 (CDK1) and CDC20 interact with CCNB1 (cyclin B1) [25]. CDC2 interacts with CCNB1 (cyclin B1) to form a complex [22] and this interaction was modeled on a demonstrated interaction between monkey CDC2 and human CCNB1 [20].<br>It is suggested that CDC2 may relate to CDC20 through CCNB1.
-CKS2(t)-CDKN1A(t) -BUB1B(t)=>- CDC25B(t+1)	Predictive	[18], [KEGG hsa:4110 (cell cycle)]	Both CDC25B and CDKN1A intereact with CDC2[18]. The interaction between CDKN1A (p21) and CDC2 was modeled on a demonstrated interaction between human p21 and hamster CDC2 [18]. And in the map hsa:4110 (cell cycle) from KEGG, CDC25B can dephosphorylate CDC2.<br>It is suggested that CDKN1A may relate to CDC25B through CDC2.

-STK15(t)=>-CKS2(t+1)	Predictive	[9, 12]	Demetrick and copateneners found that “The human cdk2/cyclin A kinase complex is a key regulator of the events of S phase. This complex contains several proteins involved in regulating its catalytic activity, including one or more of the CKS proteins, which have recently been shown to inhibit the activation of the cdk2 kinase” [12].<br>Crane and copartners found that “Cdh1 (CDC2)-activated form of the APC/C is responsible for destruction of Aur-A (STK15) during the somatic cell cycle in vivo.” [9].<br>It is suggested that STK15 may relate to CKS2 through CDC2.
-CCNE1(t)-STK15(t) =>+CDC25A(t+1)	Predictive	[9], [KEGG hsa:4110 (cell cycle)]	Crane and copartners found that “Cdh1 (CDC2)-activated form of the APC/C is responsible for destruction of Aur-A (STK15) during the somatic cell cycle in vivo.” [9]. In the map hsa:4110 (cell cycle) from KEGG, CDC25A can dephosphorylate CDC2.<br>It is suggested that STK15 may relate to CDC25A through CDC2.
-CDKN1A(t)-BRCA1(t-2) =>+CDKN1A(t+1)	Predictive	[23, 26-28]	BRCA1 physically associates with p53 and stimulates its transcriptional activity [28]. P53 is the transcription factor of CDKN1A[23, 26, 27]. It is suggested that BRCA1 may relate to CDKN1A through p53.<br>This regulation also implies CKS2 itself may regulate transcription.
-CDC2(t)=>-CDC2(t+1)	New hypothetical		This regulation also implies CDC2 itself may regulate transcription.
-CDKN1A(t)+CDC20(t-3) =>+CDKN1A(t+1)<br>(2 same regulations in results)	New hypothetical		This regulation also implies CDKN1A itself may regulate transcription.
-CKS2(t)-CDC20(t-3) =>+CDC20(t+1)	New hypothetical		This regulation also implies CDC20 itself may regulate transcription.
+BUB1B(t) =>-E2F1(t+1)	New hypothetical		There aren’t any evidences to support this regulation up to date.
-STK15(t)-CCNF(t) =>-PLK(t+1)<br>(4 same regulations in results)	New hypothetical		There aren’t any evidences to support this regulation up to date.
-STK15(t)-CKS2(t-4) =>+PLK(t+1)	New hypothetical		There aren’t any evidences to support this regulation up to date.
+CDC2(t)-BUB1B(t) =>-CCNF(t+1)	New hypothetical		
-CDC2(t)+CDC25C(t) =>+CCNF(t+1)	New hypothetical		
+CDC2(t)=>+CCNF(t+1)<br>(2 same regulations in results)	New hypothetical		
-CKS2(t)+PCNA(t-1) =>+CDC25B(t+1)	New hypothetical		
-CKS2(t)-CDC20(t-2) =>+CDC25B(t+1)	New hypothetical		

-CKS2(t)-CDC20(t-4) =>+CDC25B(t+1)	New hypothetical
+CCNE1(t)+TYMS(t) =>+CDC20(t+1)	New hypothetical
-CCNF(t) -PCNA(t) =>+CKS2(t+1)	New hypothetical
-CCNE1(t)-CCNE1(t) +CDC20(t)=>-CDC25A(t+1)	New hypothetical
-CCNE1(t)-BUB1B(t-1) =>+CDC25A(t+1)	New hypothetical
