# Supplementary material for: Discovery of time-delayed gene regulatory networks based on temporal gene expression profiling
Source: BMC Bioinformatics. 2006 Jan 18;7:26. doi: 10.1186/1471-2105-7-26 (PMC1386718; doi:10.1186/1471-2105-7-26)
Supplement: Additional File 5 — Modes of the gene-gene relationships for Hela cell cycling. [file 1471-2105-7-26-S5.pdf]

Additional file 5 – Modes of the gene-gene relationships for Hela cell cycling.

— Additional supporting analyses for the article: Xia Li, Shaoqi Rao, Wei Jiang, Chuanxing Li, Yun Xiao, Zheng Guo, Qingpu Zhang, Lihong Wang, Lei Du, Jing Li, Li Li, Tianwen Zhang and Qing K. Wang: **Discovery of time-delayed gene regulatory networks based on temporal gene expression profiling**. *BMC Bioinformatics* 2006, **7**.

Background

In the additional file, we describe the brief results of our recent study aimed at characterizing the modes of gene regulations: parallel, time-shifted and inverted, as shown in Figure S1.

Materials

We analyzed five time-series datasets that were obtained from Hela S3 cells. These public datasets are available at http://genome-www.stanford.edu/Human-CellCycle/HeLa. Cells were arrested by one of the following ways: double thymidine blocking, thymidine-nocodazole blocking and mitotic shake-off. By using three different synchronization methods in five independent experiments, Whitfield [1] identified >850 genes that are periodically expressed in cells.

Results and Discussions

We used a local alignment algorithm [2] to determine the modes of gene-gene expression relations: parallel (also called correlated), time-shifted (or time-delayed) and inverted expression relationships. Then, we compared our data with the list of periodically expressed genes identified previous by [1]. We found that the relationships for the gene pairs that are expressed in parallel or time-shifted manner are only obvious in the same or neighbouring cell cycle phase (Figure S2 A, B). Nevertheless, for the gene pairs that are in the inverted relationship, their transcriptional activities span at least one phase apart (Fig S2, C).

Therefore, we concluded that the traditional static networking approaches (i.e. without modelling the time-delayed effects) are limited for analyzing time-series data. On the other hand, the time-delayed gene networking (e.g. TdGRN) is both highly demanding for elucidating the time-delayed mechanisms, and also expected to offer maximal power to define the regulations for different modes of gene relations.

References

1. Whitfield ML, Sherlock G, Saldanha AJ, Murray JI, Ball CA, Alexander KE, Matese JC, Perou CM, Hurt MM, Brown PO *et al*: **Identification of genes periodically expressed in the human cell cycle and their expression in tumors**. *Mol Biol Cell* 2002, **13**(6):1977-2000.
2. Qian J, Dolled-Filhart M, Lin J, Yu H, Gerstein M: **Beyond synexpression relationships: local clustering of time-shifted and inverted gene expression profiles identifies new, biologically relevant interactions**. *J Mol Biol* 2001, **314**(5):1053-1066.

Figure Legends

Figure S1 - Three common modes of gene-gene relationships

Figure S2 - Network views of three expression relationships for Hela cell cycling
Each node represent a gene in the figure, pair of genes linked by line signify significant expression relationship. Blue nodes represent that the periodically expressed genes peak in G1/S phase, nacarat nodes represent that the periodically expressed genes peak in S phase, grass green nodes represent that the periodically expressed genes peak in G2 phase, yellow nodes represent that the periodically expressed genes peak in G2/M phase, fleet purple nodes represent that the periodically expressed genes peak in M/G1 phase.

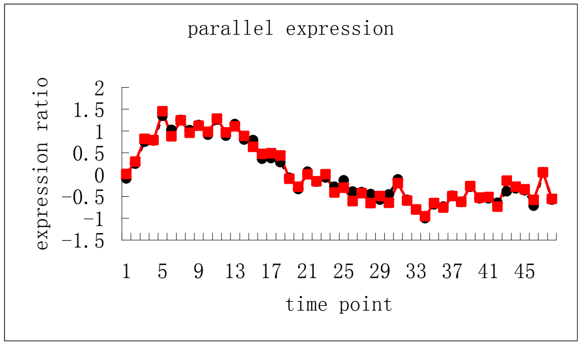

A Parallel expression

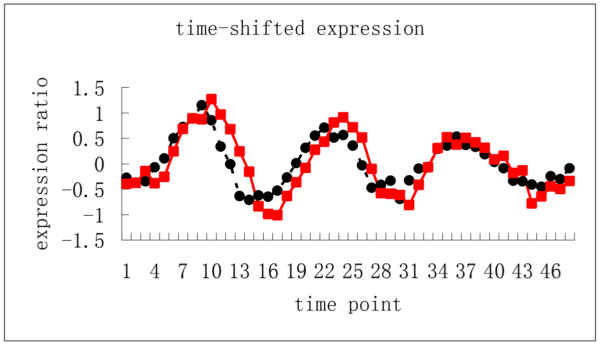

B Time-shifted expression

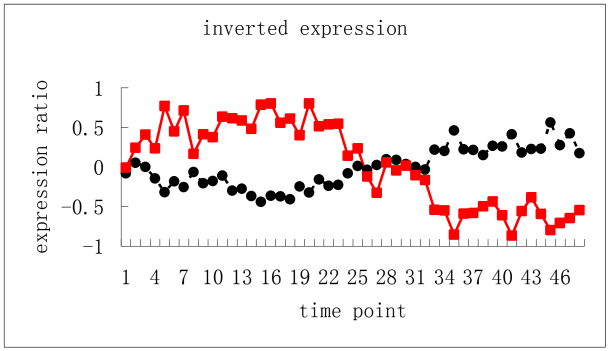

C Inverted expression

Figure S1 Three common modes of gene-gene relationships

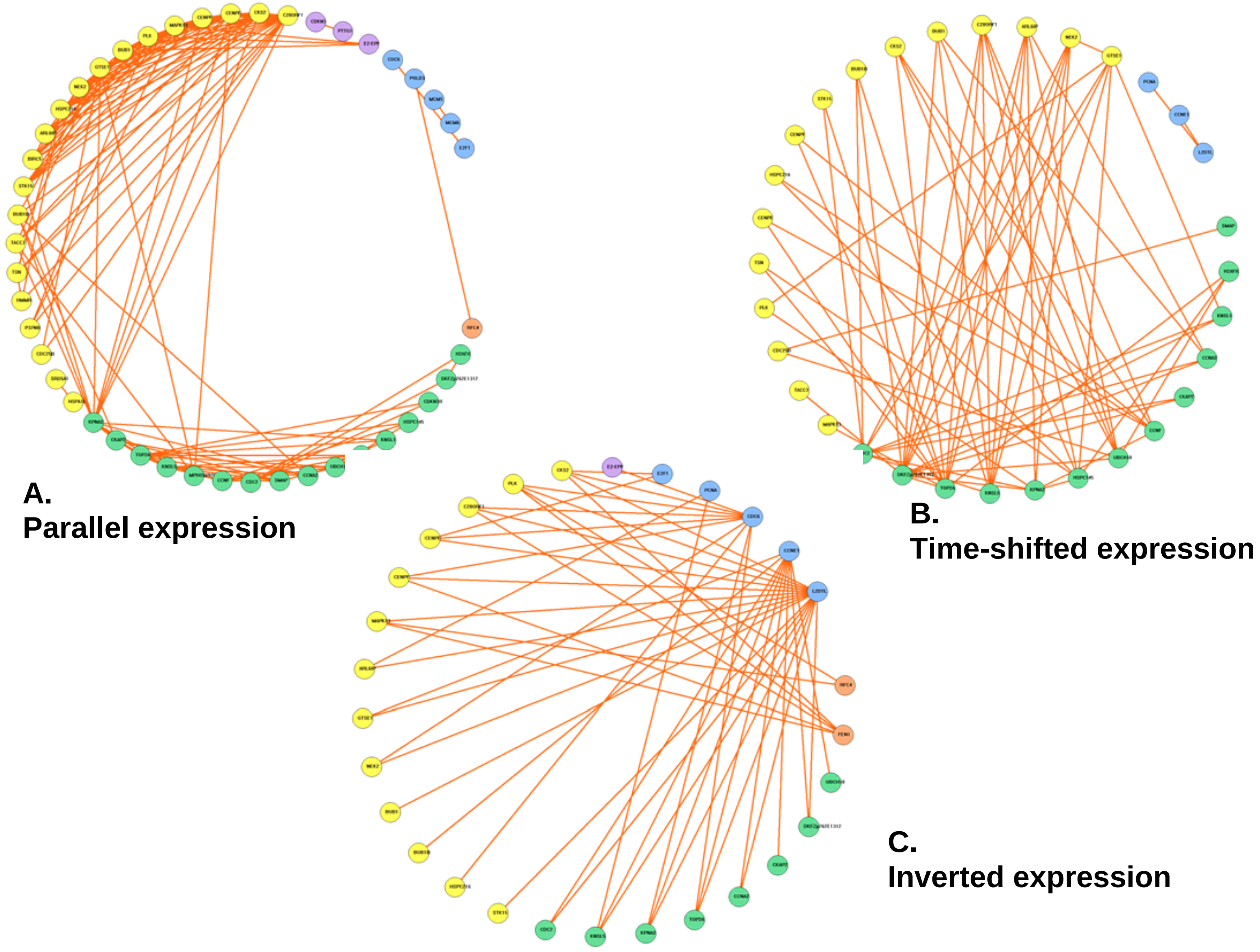

Figure S2 Network views of three expression relationships for Hela cell cycling
