# Supplementary material for: Discovery of time-delayed gene regulatory networks based on temporal gene expression profiling
Source: BMC Bioinformatics. 2006 Jan 18;7:26. doi: 10.1186/1471-2105-7-26 (PMC1386718; doi:10.1186/1471-2105-7-26)
Supplement: Additional File 6 — The empirical accuracy thresholds (α) for each yeast gene at each delayed time point (T) in three estimations (cdc15, cdc28 and α-factor), corresponding to a point-wise Type I error of 0.05. [file 1471-2105-7-26-S6.pdf]

Additional file 6 (Table S5)– The empirical accuracy thresholds (α) for each yeast gene at each delayed time point (*T*) in three estimations (*cdc28, α-factor* and *cdc15*), corresponding to a point-wise Type I error of 0.05.

Gene	*T*=1			*T*=2			*T*=3			*T*=4			*T*=5		
	cdc28	*α-factor*	*cdc15*	*cdc28*	*α-factor*	*cdc15*	*cdc28*	*α-factor*	*cdc15*	*cdc28*	*α-factor*	*cdc15*	*cdc28*	*α-factor*	*cdc15*
CLN1	0.688	0.706	0.683	0.667	0.688	0.676	0.714	0.667	0.704	0.692	0.714	0.716	0.750	0.692	0.756
CLN2	0.750	0.706	0.696	0.733	0.688	0.691	0.786	0.733	0.694	0.769	0.714	0.717	0.750	0.769	0.685
CLN3	0.688	0.706	0.691	0.733	0.688	0.667	0.714	0.733	0.704	0.769	0.714	0.743	0.750	0.692	0.784
CLB1	0.688	0.706	0.671	0.733	0.688	0.671	0.714	0.667	0.698	0.692	0.714	0.694	0.667	0.692	0.740
CLB2	0.750	0.706	0.641	0.733	0.750	0.674	0.750	0.667	0.722	0.692	0.714	0.697	0.667	0.692	0.713
CLB4	0.688	0.706	0.677	0.733	0.688	0.694	0.714	0.667	0.745	0.692	0.714	0.709	0.750	0.692	0.708
CLB5	0.688	0.706	0.665	0.733	0.688	0.694	0.714	0.667	0.682	0.692	0.714	0.727	0.667	0.692	0.734
CLB6	0.688	0.706	0.631	0.667	0.688	0.685	0.714	0.733	0.708	0.692	0.714	0.716	0.750	0.692	0.718
MCM1	0.688	0.706	0.694	0.733	0.688	0.706	0.714	0.667	0.694	0.692	0.714	0.694	0.667	0.692	0.817
SIC1	0.688	0.706	0.713	0.733	0.750	0.777	0.714	0.733	0.730	0.692	0.714	0.694	0.667	0.692	0.712
SWI6	0.688	0.706	0.664	0.733	0.688	0.665	0.714	0.667	0.701	0.692	0.714	0.704	0.750	0.692	0.694
CDC28	0.688	0.706	0.664	0.733	0.688	0.725	0.714	0.733	0.689	0.692	0.714	0.729	0.750	0.692	0.744
CDC53	0.750	0.706	0.671	0.800	0.688	0.699	0.786	0.700	0.752	0.846	0.714	0.716	0.917	0.731	0.717
MBP1	0.688	0.706	0.680	0.733	0.688	0.718	0.714	0.733	0.733	0.769	0.786	0.716	0.750	0.769	0.764
CDC34	0.688	0.706	0.680	0.667	0.688	0.678	0.714	0.733	0.703	0.692	0.714	0.710	0.667	0.769	0.726
SWI5	0.750	0.706	0.696	0.733	0.719	0.737	0.786	0.733	0.738	0.769	0.714	0.697	0.833	0.769	0.707
SKP1	0.688	0.706	0.673	0.667	0.688	0.701	0.714	0.733	0.689	0.692	0.714	0.730	0.750	0.769	0.773
SWI4	0.688	0.647	0.641	0.667	0.688	0.694	0.714	0.667	0.707	0.692	0.714	0.728	0.750	0.692	0.694
CDC20	0.688	0.706	0.688	0.700	0.688	0.707	0.714	0.733	0.697	0.692	0.714	0.702	0.750	0.692	0.712
HCT1	0.688	0.676	0.698	0.667	0.688	0.681	0.714	0.733	0.677	0.692	0.714	0.744	0.750	0.769	0.728
