# Supplementary material for: Discovery of time-delayed gene regulatory networks based on temporal gene expression profiling
Source: BMC Bioinformatics. 2006 Jan 18;7:26. doi: 10.1186/1471-2105-7-26 (PMC1386718; doi:10.1186/1471-2105-7-26)
Supplement: Additional File 7 — The empirical accuracy thresholds (α) for each human cancer gene at each delayed time point (T) in three estimations (Thy-Thy2, Thy-Noc and Thy-Thy3), corresponding to a point-wise Type I error of 0.05. [file 1471-2105-7-26-S7.pdf]

Additional file 7 (Table S6)– The empirical accuracy thresholds (α) for each human cancer gene at each delayed time point (T) in three estimations (*Thy-Thy2*, *Thy-Noc* and *Thy-Thy3*), corresponding to a point-wise Type I error of 0.05.

Gene name	*T*=1			*T*=2			*T*=3			*T*=4			*T*=5		
	Thy-Thy2	*Thy-Noc*	*Thy-Thy3*	*Thy-Thy2*	*Thy-Noc*	*Thy-Thy3*	*Thy-Thy2*	*Thy-Noc*	*Thy-Thy3*	*Thy-Thy2*	*Thy-Noc*	*Thy-Thy3*	*Thy-Thy2*	*Thy-Noc*	*Thy-Thy3*
PCNA	0.750	0.667	0.590	0.727	0.677	0.548	0.700	0.688	0.614	0.778	0.667	0.603	0.875	0.643	0.614
NPAT	0.750	0.667	0.592	0.773	0.647	0.589	0.800	0.688	0.564	0.778	0.633	0.619	0.813	0.714	0.583
E2F1	0.708	0.694	0.594	0.727	0.706	0.591	0.800	0.719	0.591	0.667	0.733	0.607	0.750	0.714	0.594
CCNE1	0.750	0.694	0.581	0.727	0.647	0.586	0.750	0.688	0.624	0.778	0.733	0.578	0.750	0.714	0.591
CDC25A	0.750	0.667	0.590	0.818	0.706	0.600	0.800	0.688	0.596	0.778	0.700	0.594	0.875	0.714	0.603
CDKN1A	0.750	0.722	0.595	0.818	0.735	0.587	0.800	0.719	0.591	0.889	0.733	0.628	0.750	0.786	0.626
BRCA1	0.750	0.667	0.592	0.727	0.647	0.604	0.800	0.688	0.600	0.778	0.700	0.604	0.750	0.679	0.597
DHFR	0.708	0.667	0.583	0.727	0.706	0.601	0.700	0.688	0.616	0.778	0.733	0.606	0.875	0.714	0.608
TYMS	0.750	0.667	0.594	0.818	0.677	0.614	0.800	0.688	0.612	0.778	0.733	0.570	0.750	0.714	0.569
CCNF	0.667	0.667	0.593	0.727	0.706	0.577	0.800	0.688	0.587	0.778	0.733	0.631	0.750	0.643	0.585
CCNA2	0.750	0.722	0.646	0.727	0.706	0.583	0.800	0.688	0.564	0.778	0.667	0.592	0.750	0.714	0.610
CDC20	0.708	0.667	0.595	0.773	0.706	0.586	0.800	0.688	0.596	0.778	0.733	0.589	0.750	0.750	0.592
STK15	0.750	0.722	0.600	0.727	0.647	0.597	0.750	0.688	0.567	0.778	0.667	0.591	0.750	0.714	0.581
BUB1B	0.667	0.667	0.598	0.727	0.706	0.608	0.700	0.688	0.612	0.778	0.733	0.629	0.750	0.714	0.597
CKS2	0.708	0.667	0.601	0.773	0.706	0.593	0.800	0.688	0.587	0.778	0.733	0.591	0.813	0.714	0.614
CDC25C	0.708	0.667	0.598	0.727	0.706	0.590	0.800	0.688	0.596	0.778	0.667	0.618	0.750	0.714	0.563
PLK	0.750	0.694	0.601	0.727	0.647	0.612	0.750	0.688	0.567	0.778	0.733	0.584	0.750	0.714	0.582
CCNB1	0.750	0.667	0.592	0.727	0.647	0.592	0.700	0.688	0.612	0.833	0.667	0.602	0.813	0.643	0.606
CDC25B	0.667	0.694	0.584	0.727	0.706	0.630	0.800	0.688	0.600	0.778	0.733	0.616	0.750	0.714	0.587
CDC2	0.708	0.667	0.588	0.727	0.706	0.579	0.700	0.688	0.614	0.778	0.733	0.571	0.750	0.714	0.577
